# Natural Rubrolides and Their Synthetic Congeners as Inhibitors of the Photosynthetic Electron Transport Chain

**DOI:** 10.1021/acs.jnatprod.4c00714

**Published:** 2024-09-06

**Authors:** Milandip Karak, Jaime A. M. Acosta, Héctor F. Cortez-Hernandez, Johnny L. Cardona, Giuseppe Forlani, Luiz C. A. Barbosa

**Affiliations:** ‡ Department of Chemistry, Universidade Federal de Minas Gerais, Av. Pres. Antônio Carlos, 6627, Campus Pampulha, CEP 31270-901 Belo Horizonte, MG, Brazil; § School of Chemical Technology, Faculty of Technology, Universidad Tecnológica de Pereira, Carrera 27 #10-02, Barrio Álamos, Código, 660003 Pereira, Risaralda, Colombia; ¶ Department of Life Science and Biotechnology, Università di Ferrara, via L. Borsari 46, I-44121 Ferrara, Italy

## Abstract

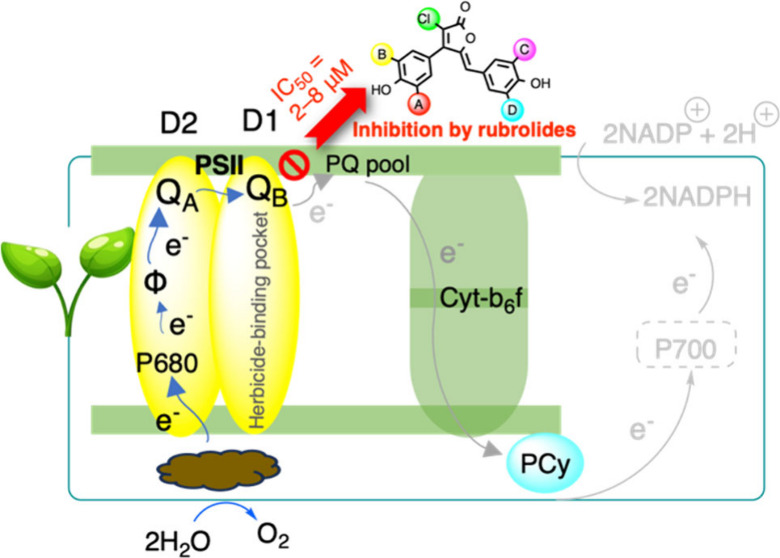

Rubrolides are a family of naturally occurring 5-benzylidenebutenolides, which generally contain brominated phenol groups, and nearly half of them also present a chlorine attached to the butenolide core. Seven natural rubrolides were previously synthesized. When these compounds were tested against the model plant *Raphanus sativus*, six were found to exert a slight inhibition on plant growth. Aiming to exploit their scaffold as a model for the synthesis of new compounds targeting photosynthesis, nine new rubrolide analogues were prepared. The synthesis was accomplished in 2–4 steps with a 10–39% overall yield from 3,4-dichlorofuran-2(5*H*)-one. All compounds were evaluated for their ability to inhibit the whole Hill reaction or excluding photosystem I (PSI). Several natural rubrolides and their analogues displayed good inhibitory potential (IC_50_ = 2–8 μM). Molecular docking studies on the photosystem II-light harvesting complex II (PSII-LHCII supercomplex) binding site were also performed. Overall, data support the use of rubrolides as a model for the development of new active principles targeting the photosynthetic electron transport chain to be used as herbicides.

Modern agriculture heavily relies on synthetic herbicides to control weeds and enhance crop productivity.^1^ However, due to the increasing emergence of herbicide-resistant biotypes, a continuous development of new active principles is needed.^2^ Recent studies have suggested that synthetic chemistry approaches tend to produce large chemical libraries that have limited effects on a small number of target sites. In contrast, natural products and natural-product-like herbicides have a unique mode of action^1a,2b,3^ and usually show environmentally friendly properties, as they break down more quickly in soil compared to synthetic herbicides.^4^ Moreover, the structural diversity of natural compounds provides an opportunity to discover new targets of herbicide action.^5^ Several herbicides that are currently in use target the photosynthetic process by interfering with different aspects of the electron transport chain and ATP synthesis. They can be categorized into five main types: a) electron transport inhibitors, b) uncouplers, c) energy transfer inhibitors, d) inhibitory uncouplers, or e) electron acceptors.^6^ Therefore, targeting photosynthesis continues to be a promising approach for developing herbicides.^7^

During recent years, several natural butenolides have been found to possess herbicidal properties.^8^ For instance, a smoke-derived trimethylbutenolide has been extensively studied due to its potent activity against seed germination.^8c,8d^ On the other hand, smoke-derived 3-methyl-2*H*-furo[2,3-*c*]pyran-2-one has shown positive effects on seed germination and seedling vigor, increasing both the level and rate of seed germination and widening the range of environmental conditions under which germination can occur.^8a,9^

Research in our laboratories aims to develop new agrochemicals for weed control by synthesizing biologically active natural product analogues.^10^ In previous studies, we synthesized several compounds using nostoclides as models, but their activity was found to be limited by poor H_2_O solubility.^10,10c,10d,^ We then turned our attention to rubrolides and prepared several new analogues that are effective inhibitors of the Hill reaction.^10e,10h,10i^ These rubrolide analogues were found to be promising candidates for developing new active principles targeting photosynthesis due to their good H_2_O solubility. A Quantitative Structure–Activity Relationship (QSAR) analysis showed that the effectiveness of these compounds is associated with the presence of electron-withdrawing groups (EWGs) at the benzylidene unit.^10e^ Cyclopent-4-ene-1,3-diones, which can be directly obtained from certain rubrolide analogues, were found to be even more active than rubrolides, and as potent as the commercial herbicide diuron.^10g^ We demonstrated that these compounds inhibit the photosynthetic electron transport by binding the D1 protein, making them promising candidates for developing novel herbicides targeting photosynthesis.^10g^

While most of the active rubrolide analogues have a 3-bromo substituent, none of the natural rubrolides and their 3-chloro analogues have been investigated as photosynthesis inhibitors. Herein, we report the synthesis of new 3-chlororubrolide analogues and evaluate them, alongside natural rubrolides **1**–**7**, and compounds **8**–**10** for their ability to inhibit the Hill reaction.
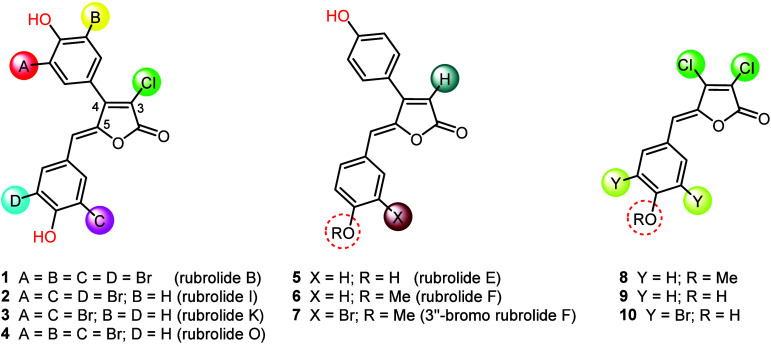


A detailed investigation was carried out using molecular docking studies to examine the binding sites of the PSII-LHCII supercomplex, providing additional support for the experimental results. Furthermore, we assessed the effects of natural rubrolides **1**–**7** on the growth of the model species *Raphanus sativus*.

## RESULTS AND DISCUSSION

### Natural Rubrolides Exert a Mild but Significant Inhibition of Plant Growth

In our previous work, we synthesized seven natural rubrolides (**1**–**7**) starting from commercially available 3,4-dichlorofuran-2(5*H*)-one.^11^ Rubrolides B, I, K, and O (**1**–**4**) were synthesized in 3–4 steps, with overall yields ranging from 35–41%. Key synthetic steps included a site-selective Suzuki cross-coupling, a vinylogous aldol condensation, and a late-stage bromination, which enabled regioselective functionalization of the aromatic rings.^11a^ Additionally, our hydrodehalogenation protocol was employed to synthesize rubrolides E and F, and 3″-bromorubrolide F (**5–7**) in 4–5 steps and overall yields of 54–65%.^11b^

**Figure 1:**
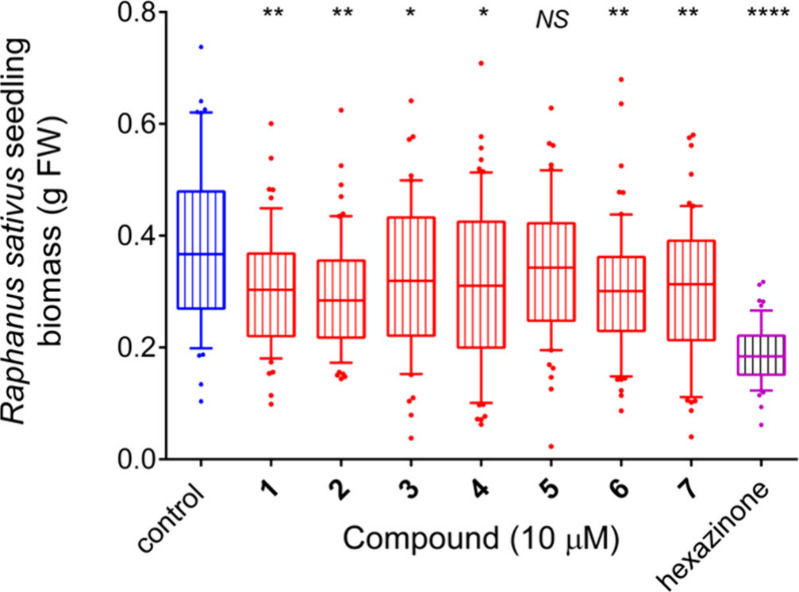
Effect of natural rubrolides **1**–**7** on the growth of the model plant *Raphanus sativus*. Seeds were sown in the presence of a given compound at a concentration of 10 μM. Controls received the same volume of DMSO (0.1%) used to dissolve the rubrolides. The commercial herbicide hexazinone was also included, as a positive control. Growth was measured when untreated plants reached the three-leaf stage, 3 weeks after sowing. Results were analyzed using Prism 6 for Windows by means of the Kolmogorov–Smirnov normality test (*P* = 0.05), and then subjected to 1-way ANOVA. A Holm-Sidak’s multiple comparisons test showed significant even if slight growth inhibition in all samples treated with rubrolides, with the only exception of compound **5** (*P < 0.05, **P < 0.01, ****P < 0.0001). Results were plotted as a box and whiskers plot showing 10–90th percentiles.

The ability of these compounds to interfere with plant growth was evaluated by measuring the growth of *Raphanus sativus* seedlings that had been sown in the presence of a given rubrolide. Results (Figure 1) showed that, with the only exception of rubrolide E (**5**), all these natural compounds exert a mild, yet significant phytotoxicity at 10 μM. Data are consistent with a recent study on rubrolides C, E (**5**), and F (**6**), which were found to inhibit rapeseed growth in greenhouse treatments.^12^ In that case, compound **5** was also found inhibitory, but at a 20-fold higher concentration (200 μM).

### Some New Rubrolide Analogues Were Synthesized with Satisfying Yield

In our approach toward the synthesis of rubrolide analogues **12**–**18** (Table 1), we began with commercially available 3,4-dichlorofuran-2(5*H*)-one. To introduce the aryl group at the 4-position in 3,4-dichlorobutenolide, a selective Suzuki coupling was utilized with a palladium catalyst, triphenylphosphine, and cesium fluoride conditions that have being previously developed.^11b^ The reaction produced the desired 4-aryl-3-chloro intermediates **11a**–**d** in 62–85% yields using several phenylboronic acids. We prepared four compounds with varying substituents on the phenyl ring: compound **11a** with an unsubstituted phenyl ring, compound **11b** with 5-chloro-2-methoxy substitutions, compound **11c** featuring an electron-withdrawing fluorine atom at position 4, and compound **11d** with an additional electron-donating methoxy group at position 2. The reactions exhibited high regioselectivity, as observed in similar cases,^10e,10i^ and the structures of all new products were confirmed through spectroscopic analysis (see the ESI).

**Table 1:**
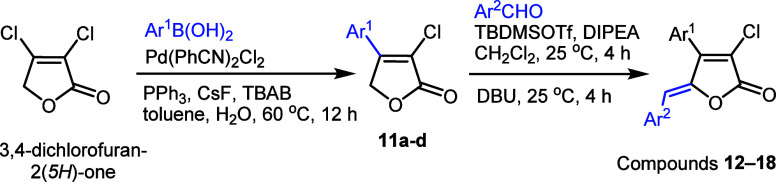

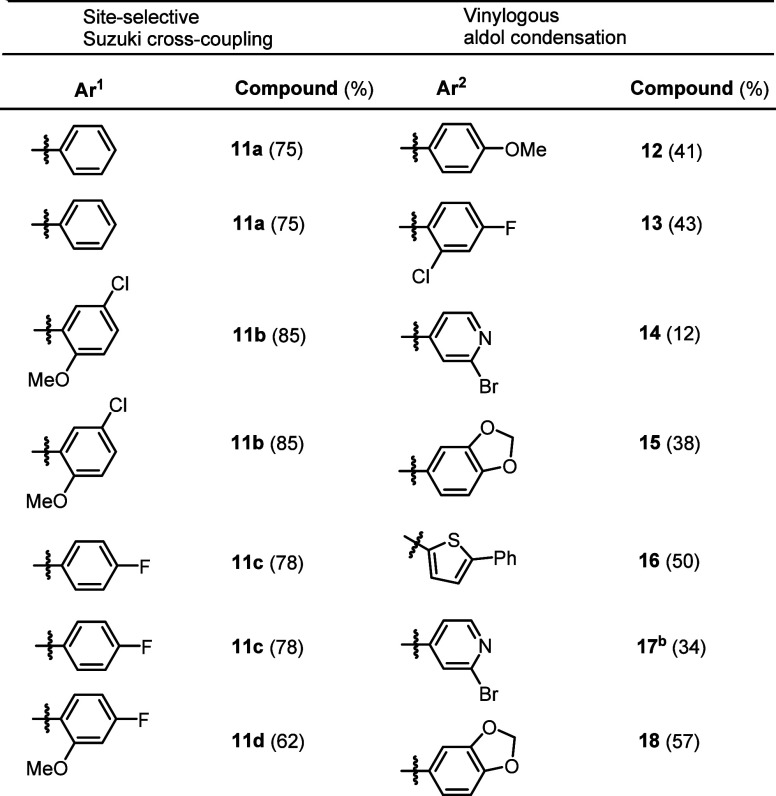
Preparation of New Rubrolide Analogues **12**–**18**^a^

a All yields are isolated yield.

b Compound **17** was isolated as the mixture of *Z*/*E* (0.74/0.26) isomers.

Next, compounds **11a**–**d** were converted into the benzylidenes **12**–**18**, using a similar method to the one earlier employed for synthesizing nostoclide and rubrolide analogues.^10c,10e^ The two-step, one-pot process initially involved a vinylogous aldol condensation between intermediates **11a**–**d** and corresponding aldehydes, followed by the elimination of *tert*-butyldimethylsilanol. To achieve this, lactones **11a**–**d** were subjected to treatment with *tert*-butyldimethylsilyltriflate and diisopropylethylamine, which yielded the corresponding silyl ethers. These intermediates were not isolated, but their formation has been previously proven.^10a^ In the present study, they were directly reacted with 1,8-diazabicyclo[5.4.0]undec-7-ene, leading to the formation of desired compounds **12**–**18** in a stereoselective manner (*Z*-isomer only in most cases). The yields of the reaction ranged from 12% to 57%, as shown in Table 1.

**Scheme 1:**
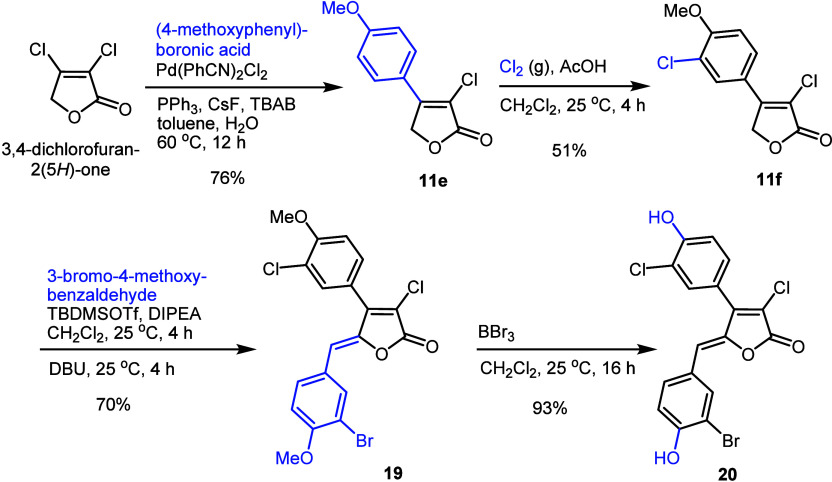
Preparation of the Chloro-Analogue of Rubrolide K **20**

Finally, the synthesis of a chloro-analogue of rubrolide K **20** was completed as outlined in Scheme 1. Starting from 3,4-dichlorofuran-2(5*H*)-one and 4-methoxyphenylboronic acid, compound **11e** was obtained in 76% yield by using a procedure similar to that described for compounds **11a**–**d**. Subsequently, chlorination of compound **11e** was carried out using freshly prepared chlorine gas and acetic acid as an additive in anhydrous CH_2_Cl_2_, resulting in a 51% yield of the monochlorinated product **11f**. The next step involved a vinylogous aldol condensation of **11f** with 3-bromo-4-methoxybenzaldehyde, which produced compound **19** exclusively as the *Z*-isomer in 70% yield. The ensuing removal of the methyl groups from compound **19** using boron tribromide afforded compound **20** in overall 25% yield. It is worth noting that a different approach was previously used to synthesize compound **20**.^13^

### Most Natural Rubrolides and Some of Their Analogues Are Inhibitors of the Photosynthetic Electron Transport Chain

The ability of natural rubrolides and their synthetic analogues to interfere with the chloroplast electron transport chain was evaluated by measuring the Hill reaction in thylakoid membranes isolated from spinach leaves. The results were summarized as the concentrations able to inhibit by 50% the rate measured in untreated controls (IC_50_, Table 2). Rubrolides displayed moderate to good inhibitory potential when compared to that of the commercial herbicide diuron. Interestingly, the only natural rubrolide that was found ineffective over the whole range of concentrations tested (1–100 μM), namely rubrolide E (**5**), is the same that had been found ineffective against the growth of radish seedlings.

**Table 2:** Concentrations of Natural Rubrolides **1**–**7**, Compounds **8**–**10**, and Rubrolide Analogues **12**–**20** Were Able to Inhibit by 50% the Light-Dependent Ferricyanide Reduction in Isolated Spinach Thylakoids

Compound	IC_50_ (μM)	Compound	IC_50_ (μM)
**1** rubrolide B	8.62 ± 1.89	**12**	257 ± 273
**2** rubrolide I	5.54 ± 0.98	**13**	35.9 ± 7.7
**3** rubrolide K	8.00 ± 2.05	**14**	61.4 ± 54.2
**4** rubrolide O	4.37 ± 0.80	**15**	1620 ± 340
**5** rubrolide E	n.e.^a^	**16**	266 ± 272
**6** rubrolide F	29.3 ± 4.80	**17**	n.e.^a^
**7** 3″-bromorubrolide F	56.6 ± 15.2	**18**	438 ± 260
**8**	n.e.	**19**	n.e.
**9**	n.e.	**20**	4.97 ± 1.05
**10**	2.88 ± 1.50	Diuron	0.27 ± 0.01

a n.e. – not effective over the whole range of concentrations tested (0.05–100 μM). Each treatment was performed in triplication; IC_50_ and confidence limits were calculated by nonlinear regression analysis using GraphPad Prism 6.

An initial Structure–Activity Relationship (SAR) analysis suggested a significant impact of the presence of the hydroxy group on the resulting activity. For example, compound **20**, featuring hydroxy groups, showed an IC_50_ value of 4.97 μM, whereas its methoxy counterpart, compound **19**, had no activity. Similarly, other methoxylated compounds **6**, **7**, **14**, **15**, and **18** showed IC_50_ values higher than 25 μM. Further analysis confirmed that both compounds **19** and **20** satisfy all parameters of Lipinski’s rule of five^14^ (see the ESI, Table S1). The primary difference between the two compounds was observed in their LogS values,^15^ with compound **19** showing a value of −6.29 and compound **20** a value of −5.86 (Table S1), indicating that compound **20** has better H_2_O solubility. This suggests that the presence of free hydroxy groups likely enhances H_2_O solubility, potentially facilitating access to the active site, which could be pivotal for optimizing compound design and achieving enhanced activity in this specific case.^10c^

On the other hand, the presence of halogens in the molecules emerges as another crucial determinant of activity. Compounds containing bromine and chlorine atoms, such as natural rubrolides **1**–**4**, compound **10**, and analogue **20**, exhibit noteworthy activity. The notable effects of halogens were primarily observed in the 4-aryl and 5-benzylidene positions as well as in the 3-positions on the butenolide core. Former effects are evident in compounds **5**–**9**, displaying low or no activity at all, while the latter is appreciable in compounds **5**, **8**, and **9**, which lack activity. Interestingly, replacing bromine with chlorine in the 4-aryl ring led to a significant enhancement in activity, as observed in rubrolide K (**3**) and its analogue **20**. The improved activity is likely due to the better H_2_O solubility of compound **20**, as indicated by its higher LogS value (−5.86) compared to that of rubrolide K (**3**) (−6.18) (Table S1). The SAR studies also suggest that the most efficient compounds have a higher capacity to accept electrons, particularly through reduction processes or electrophilic reaction mechanisms involving halogens.^10i^ Conversely, compounds with a heterocycle scaffold in the 5-benzylidene position (**14**–**18**) and fluorine at the 4-aryl ring (**16**–**18**) exhibit limited activity (IC_50_ > 60 μM). Notably, rubrolides I (**2**) and O (**4**) exhibited nearly double the activity of rubrolides B (**1**) and K (**3**), indicating that both the total number of halogen atoms and their specific positions are also critical factors influencing their activity. The significant activity of compound **10** can be attributed to its specific structural features: the presence of two chlorine atoms in the butanolide core, 5-benzylidene bromine substitutions, and a hydroxy group on the benzylidene aromatic ring, all of which seem crucial for enhancing its activity.

**Figure 2:**
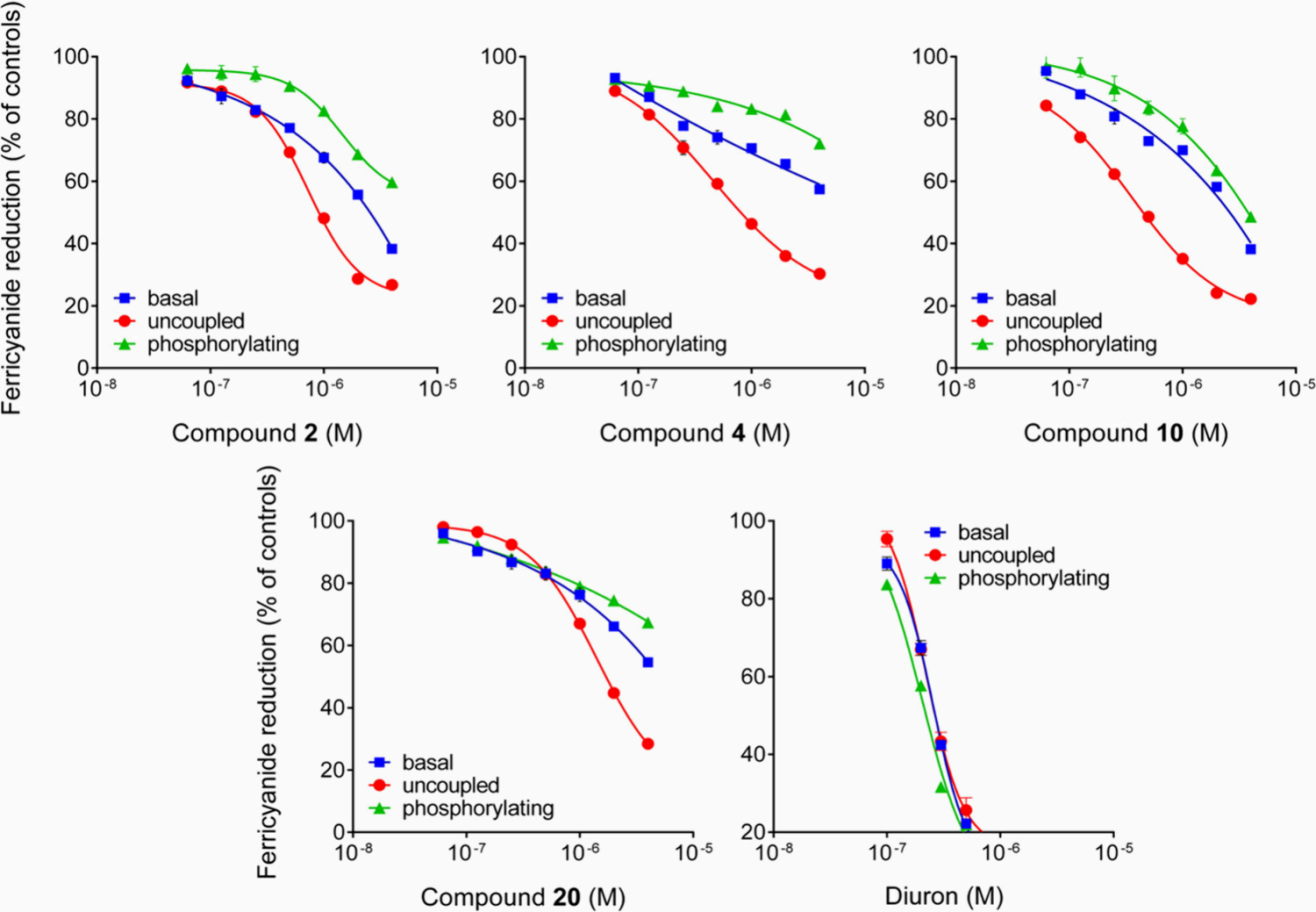
Effect of increasing concentrations of compounds **2**, **4**, **10**, and **20** on ferricyanide reduction under basal, uncoupling, or phosphorylating conditions. Diuron was included as a positive control.

In order to obtain more information about their mechanism of action, the ability of the most active compounds (**2**, **4**, **10**, and **20**) to interfere with ferricyanide reduction by isolated spinach chloroplast was measured also under uncoupling and phosphorylating conditions. Results showed in all cases a progressive reduction of the activity in the 10^–7^ to 10^–5^ M range (Figure 2), and excluded the possibility that such compounds act as energy coupling or uncoupling inhibitors, leading to a disruption in ATP synthesis.

Herbicides that interfere with the photosynthetic electron transfer effectively prevent the establishment of a transmembrane electrochemical gradient, thus impeding ATP synthesis.^16^ Substances capable of disrupting the thylakoid membranes and increasing proton permeability can cause uncoupling between phosphorylation and electron flow.^16,17^ This would result in enhanced rates of ferricyanide reduction under basal conditions, which was not the case for the examined rubrolides that should therefore be regarded as electron transport inhibitors.

**Figure 3:**
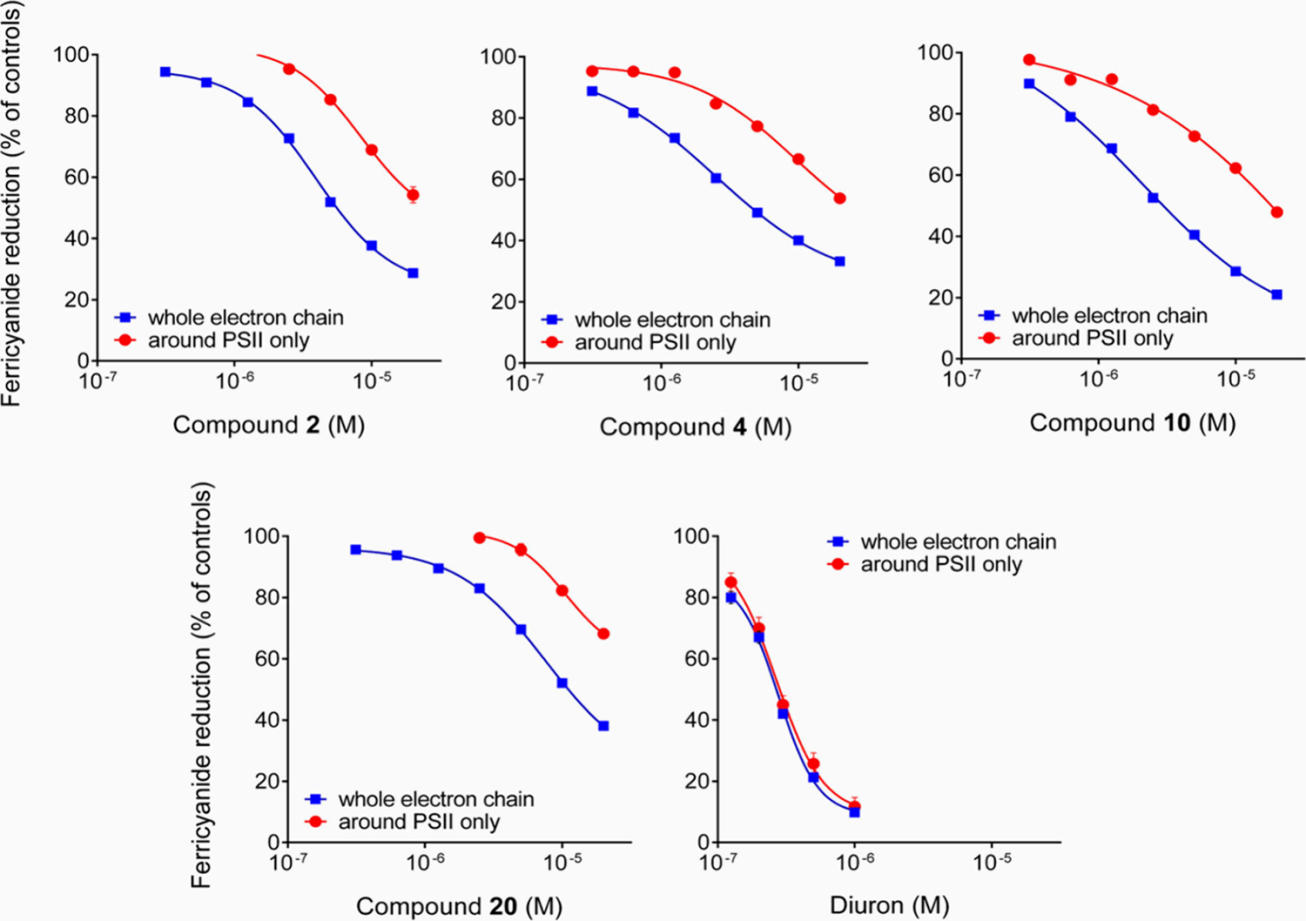
Effects of compounds **2**, **4**, **10**, and **20** and diuron on the whole photosynthetic electron transport chain and on a partial electron flow involving PSII only.

To shed further light on the mechanism of action, the effects of the most active compounds (**2**, **4**, **10**, and **20**) were evaluated also under conditions that exclude PSI. Results (Figure 3) showed that compounds are able to inhibit the electron transfer from PSII to the cytochrome b6f complex.

Contrary to diuron, which displayed a very similar effectiveness, the inhibition brought about on PSII was slightly but significantly lower than that on the whole transport chain. This could suggest the presence of other targets in the photosynthetic apparatus. However, the possibility that rubrolides may interfere with PSI is unlikely since PSI-inhibiting herbicides usually possess ionic groups. An interference with the cytochrome b6f complex cannot be completely excluded, but this difference could simply rely on the experimental conditions employed. To evaluate the effect on PSII only, uncoupled activity is blocked by the addition of the cytochrome b6f inhibitor 2,5-dibromo-6-isopropyl-3-methyl-1,4-benzoquinone, and an electronic transfer from H_2_O to ferricyanide that excludes PSI is restored through addition of phenylenediamine. This results in a much lower rate (15–20%) of ferricyanide reduction, so that higher amounts of thylakoid membranes were used for the assay, amounts that may “dilute” the effect of the compounds. A similar effect was previously noticed also in the case of other inhibitors.^10b,10e,10j^

### In Silico Docking Studies

Most commercial herbicides targeting photosynthesis act by interfering with the D1 quinone-binding protein (referred to as the QB protein).^18^ In this manner, the inhibitors block the whole electron transport process. Plastoquinone binding to QB protein involves interactions with HIS_215_ and SER_264_ residues.^18^ Based on their mode of action, PSII-inhibiting herbicides can be broadly categorized into urea/triazine-type (targeting SER_264_) and phenol-type (targeting HIS_215_) compounds.^18,19^ The latter include an aromatic hydroxy group with an electron withdrawing group (EWG) and/or halogen and/or a sterically unhindered lipophilic group, which binds to the QB-site through D1-HIS_215_.^19b^ Certain rubrolides seem to possess such properties. These analogues act as EWGs with minimal steric demands and provide the required lipophilicity commonly seen in phenol-based herbicides.^16−20^

To strengthen our conclusions, we conducted molecular docking studies on the interaction of the most active compounds (**2**, **4**, **10**, and **20**) with PSII (D1 protein), using the crystallographic structure of thylakoid membranes isolated from spinach (*Spinacia oleracea*, PDB ID: 3JCU).^21^ We used AutoDock Vina^22^ for the molecular docking calculations and performed comparative docking with SwissDock^23^ to validate the interactions.

**Figure 4:**
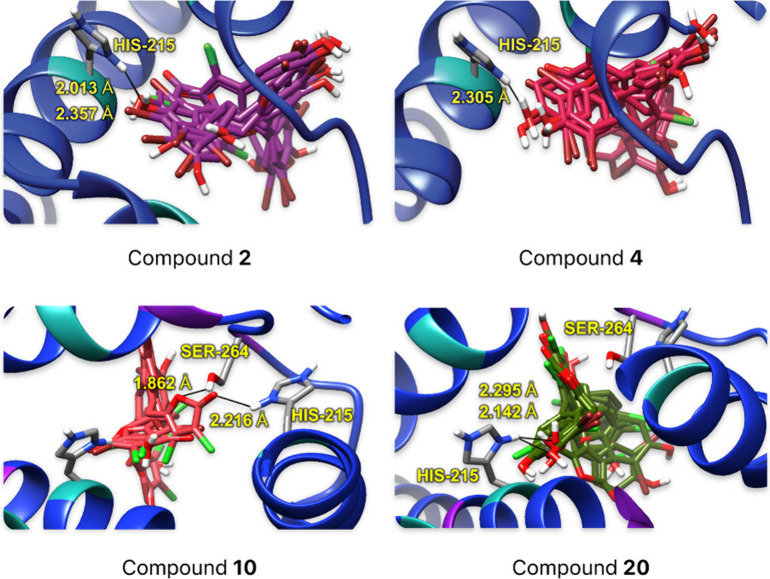
Population analysis of compounds **2**, **4**, **10**, and **20** at the pocket site in PSII (PDB ID: 3JCU)^21^

The molecular docking analysis of rubrolides revealed strong chemical interactions with the PSII (D1) binding pocket, primarily through hydrogen bonds with the HIS_215_ and SER_264_ residues. Commercial herbicides lenacil and diuron exhibited interaction energy values between −7.0 and −8.9 kcal/mol, while rubrolides and their analogues (**1**–**10** and **12**–**20**) showed values ranging from −7.0 to −10.7 kcal/mol (see the ESI; Table S2). These results suggest that the observed in vitro inhibition may be linked to the high binding affinity of rubrolides and their analogues within the PSII (D1) binding pocket. On the other hand, a population analysis was performed to assess the positioning of the most active compounds (**2**, **4**, **10**, **20**) relative to the interaction site (Figure 4). It was found that at least nine poses were in close proximity to the active site (RMSD < 5%) for each compound evaluated. This observation confirms that the active compounds have a 99% probability of interacting in the pocket site, with the most relevant interactions being with the HIS_215_ and SER_264_ residues.

**Figure 5:**
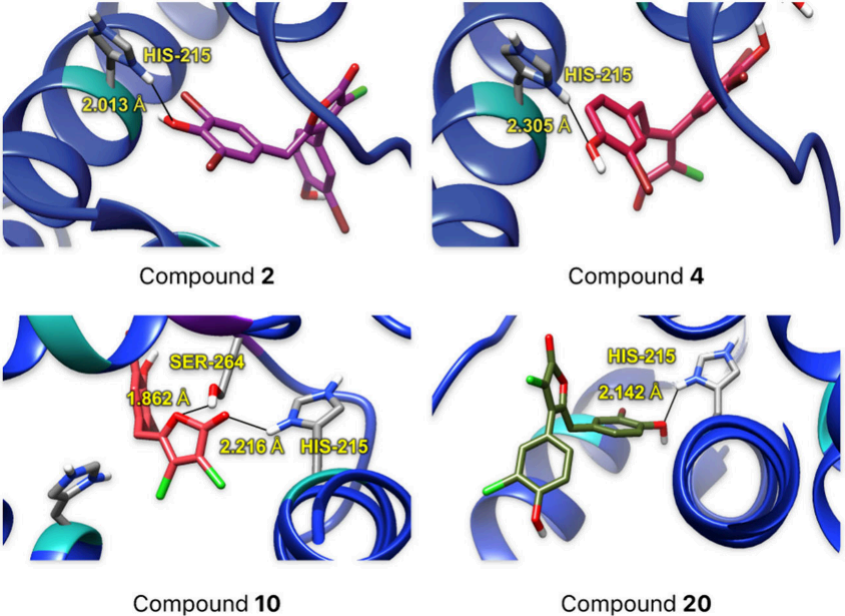
Bond distances determined between compounds **2**, **4**, **10**, and **20**, and amino acid residues at the pocket site in PSII (PDB ID: 3JCU).^21^

To further assess the nonbonding parameters affirming the interaction strength at the binding site, we investigated the presence and distances of hydrogen bonds between polar hydrogens of HIS_215_ and SER_264_ residues and compounds **2**, **4**, **10**, and **20**. These interactions mainly involved hydroxy groups of compounds **2**, **4**, and **20**, and the cyclic amino groups in the HIS_215_ residue (Figure 5). The bond distances for all tested compounds ranged from 1.8 to 2.3 Å, with donor-H···acceptor bond angles typically between 120° and 160° (see ESI; Table S3). According to the Jeffrey scale,^24^ these parameters indicate moderate hydrogen bonds. HIS_215_ emerged as the primary H-donor, forming bonds mainly with oxygen atoms of compounds such as carbonyls (in compound **10**) or hydroxy groups (in compounds **2**, **4**, and **20**). Similarly, a recent study showed that diuron forms a strong hydrogen bond between its carbonyl group and the HIS_215_ residue.^25^ Another commercial herbicide, lenacil, occupies the region near the residues HIS_215_, SER_264_, and PHE_265_ in PSII (D1).^10j,26^ Most interactions involved −N–H bond donation and −O–H acceptance from the benzylidene aromatic rings of the rubrolide moiety (as seen with compounds **2**, **4**, and **20**), while compound **10** displayed notable interactions within the pocket site, forming more hydrogen bonds within the butenolide core itself and interacting with HIS_215_ and SER_264_ residues. These findings suggest a variety of dynamic hydrogen-bond interactions at the binding site, potentially inhibiting PSII by disrupting electron transfer in plastoquinone at the QB site on the D1 protein, thereby affecting the photosynthesis mechanism.^10j^ To our knowledge, this study represents the first molecular docking analysis of rubrolides within the PSII (D1) binding site.

## Conclusion

In summary, this study demonstrated that at a concentration of 10 μM, certain natural rubrolides effectively inhibits the growth of the model plant *Raphanus sativus*. Four among natural and synthetic rubrolides were found to be considerably effective against the photosynthetic electron transport chain, with IC_50_ values ranging from 2.80 to 5.00 μM. The SAR analysis indicated that the activation of these compounds is associated with the presence of hydroxy and halogen groups as well as their good water solubility. These compounds primarily inhibit electron transfer from PSII to the cytochrome b6f complex without significantly affecting PSI, indicating their role as electron transport inhibitors rather than uncouplers or ATP synthesis inhibitors. Molecular docking studies further support this, showing that these compounds effectively bind to the PSII (D1) binding pocket and interact with the HIS_215_ and SER_264_ residues. These findings suggest that rubrolides could be promising candidates for herbicide development, owing to their targeted disruption of the photosynthetic electron transport chain.

## EXPERIMENTAL SECTION

The synthetic methods utilized in this study closely followed the procedures previously published by our research group.^11^ Our group has previously reported the complete experimental and characterization specifics of natural rubrolides B, E, F, 3″-bromo rubrolide F, I, K, and O (**1**–**7**), as well as compounds **8**–**10**, covering their synthetic procedures, physical properties, and spectroscopic data.^11^ In this article, we introduce nine new analogues **12**–**20** along with their respective preparation methods. All reactions were conducted using solvents of analytical grade. Reagents and solvents were subjected to purification and drying procedures when necessary. Air- and moisture-sensitive reactions were executed under nitrogen or argon atmospheres, within oven-dried glassware sealed with rubber septa. Sensitive liquids, solutions, and anhydrous solvents were transferred using syringes or cannulas through the rubber septa. TLC analyses were carried out on aluminum-backed precoated silica gel plates (Polygram-UV254, 0.20 mm thickness, Macherey-Nagel, 20 × 20 cm), observed under UV light (at wavelengths of 254 and 365 nm). Column chromatography was performed using silica gel (mesh size: 230–400). Melting points, reported without correction, were determined using an MQAPF-301 melting point apparatus (Microquimica, Brazil).

### General Spectroscopic Techniques

^1^H (400 MHz) and ^13^C NMR (100 MHz) spectroscopic data were acquired on a Bruker NMR spectrometer utilizing deuterated solvents (CDCl_3_, DMSO-*d*_6_, or acetone-*d*_6_), occasionally with tetramethylsilane (TMS) as an internal standard (δ = 0). The infrared spectrum was recorded on a Varian 660-IR spectrometer equipped with GladiATR scanning from 4000 to 500 cm^–1^. High-resolution mass spectrometry (HRMS) was conducted using a Bruker MicroTof (with a resolution of 10,000 fwhm) employing electrospray ionization and reported to four decimal places.

### General Synthetic Procedure for 4-Aryl-3-chloro-butenolides (**11a**–**e**)

In a 100 mL two-neck round-bottom flask, commercially available 3,4-dichlorobutenolide (2.0 g, 1.0 equiv.) was mixed with the corresponding boronic acid (3.0 equiv.), cesium fluoride (3.0 equiv.), Pd(PhCN)_2_Cl_2_ (0.05 equiv.), PPh_3_ (0.1 equiv.), Bu_4_NBr (0.1 equiv.), and 24 mL of toluene/H_2_O (2:1). The reaction mixture was degassed for 10 min under a nitrogen atmosphere and then stirred at 60 °C for 12 h. Subsequently, the sample was filtered through a Celite pad. The filtrate underwent extraction with EtOAc (3 × 50 mL), and the combined organic layer was dried over anhydrous Na_2_SO_4_ before being concentrated under vacuum. The crude product was further purified by column chromatography on silica gel, using a mixture of hexane and EtOAc as an eluent. See the ESI for compounds characterization data.

### Synthesis of 3-Chloro-4-(3-chloro-4-methoxyphenyl)furan-2(5H)-one (**11f**)

In a 50 mL two-neck round-bottom flask, compound **11e** (200 mg, 1.0 equiv.) was dissolved in anhydrous CH_2_Cl_2_ (10 mL) at room temperature, with acetic acid added as an additive. An empty balloon was then placed in the side-neck of the round-bottom flask, and freshly prepared chlorine gas, generated from potassium permanganate and concentrated HCl, was directly introduced into the stirred reaction mixture in a fume hood for 1 h at room temperature. The resulting solution was further stirred for 4 h under a chlorine balloon. After confirming the consumption of the starting material by TLC analysis, the reaction mixture was quenched with a saturated solution of Na_2_S_2_O_3_, and CH_2_Cl_2_ was subsequently evaporated under vacuum. The aqueous phase was extracted with EtOAc (3 × 15 mL), and the combined organic layers were dried over anhydrous Na_2_SO_4_, filtered, and evaporated under vacuum. The crude product was further purified by column chromatography on silica gel eluted with a mixture of hexane and EtOAc and was obtained as a white solid in 51% yield. Compound characterization data are available in the ESI.

### General Synthetic Procedure for 4-Aryl-5-benzylidene Butenolides (**12**–**19**)

A solution containing **11a**–**d**/**11f** (200 mg, 1.0 equiv.) in anhydrous CH_2_Cl_2_ (5 mL) was prepared in a 25 mL two-neck round-bottom flask. The solution was degassed for 5 min under a nitrogen atmosphere and cooled to 0 °C. Subsequently, *i*-Pr_2_NEt (3.0 equiv.) and TBDMSOTf (2.0 equiv.) were sequentially added under a nitrogen atmosphere. The reaction mixture was stirred for 30 min at 0 °C, followed by the addition of the corresponding aldehydes (1.2 equiv.). After stirring for 1 h at 0 °C, the reaction mixture was allowed to stir at room temperature for 2 h. Next, DBU (2.0 equiv.) was added, and the resulting mixture was stirred for another 4 h. After this duration, the solution was quenched with HCl (1 M, 10 mL) at 0 °C. CH_2_Cl_2_ was then removed under vacuum, and the aqueous phase was extracted with EtOAc (3 × 15 mL). The combined organic layers were washed with a saturated NaCl solution, dried over anhydrous Na_2_SO_4_, and concentrated under vacuum. Finally, the crude product was purified by column chromatography on silica gel, using a hexane/EtOAc eluent. For compound characterization, see the ESI.

### Synthesis of (*Z*)-5-(3-Bromo-4-hydroxybenzylidene)-3-chloro-4-(3-chloro-4-hydroxyphenyl)furan-2(5H)-one (**20**)

In a 50 mL two-neck round-bottom flask, a solution of compound **19** (50 mg, 1.0 equiv.) in anhydrous CH_2_Cl_2_ (15 mL) was stirred at 0 °C, while boron tribromide (1.5 equiv.) was added dropwise. The resulting mixture was then allowed to warm to room temperature and stirred for an additional 16 h. Subsequently, the reaction was quenched with an aqueous NH_4_Cl solution (20 mL), and CH_2_Cl_2_ was removed under vacuum. The aqueous phase was extracted with EtOAc (3 × 20 mL), and the organic layer was dried over anhydrous Na_2_SO_4_, filtered, and evaporated under vacuum. The crude product was purified by column chromatography on silica gel and eluted with a mixture of hexane and EtOAc (3:7 v/v), yielding compound **20** as a white solid in 92% yield.

### Evaluation of the Ability of Compounds **1**–**7** to Inhibit Plant Growth

Seeds of *Raphanus sativus* cv Scarlet Champion were surface sterilized by treatment with ethanol for 4 min and with 3% NaClO containing 0.04% Triton X-100 for 8 min under vacuum. Following extensive washing with sterile double distilled H_2_O, seeds were transferred to GA7 Magenta vessels containing 50 mL of agarized (7‰) 0.5X MS salts with 1 mL L^–1^ of Plant Preservative Mixture (Plant Cell Technology, Washington, DC), supplemented or not with the test compounds to a final concentration of 10 μM. Parallel controls received the same volume (50 μL) of DMSO. Twelve seeds were sown in each vessel, in a completely randomized design with 4 replicates. Vessels were incubated in a FOC 200IL incubator at 24 ± 0.5 °C under 150 μmol m^–2^ s^–1^ PAR with a 12:12 light:dark photoperiodic cycle. After 8 days of growth, the lid was replaced with a coupler and a second vessel, obtaining a 20 cm height vessel. Under these conditions, untreated plants reached the three-leaf stage 20 days after sowing. The fresh biomass of each seedling was measured at that point by a destructive harvest. The plant material harvested from each vessel was then combined and treated in an oven at 80 °C for 3 days for the determination of dry matter.

### Evaluation of the Activity against the Photosynthetic Electron Transport Chain

Thylakoid membranes were isolated from spinach (*Spinacea oleracea* L.) leaves. Plant material was resuspended in 5 mL g^–1^ of ice-cold 20 mM Tricine-NaOH buffer (pH 8.0) containing 10 mM NaCl, 5 mM MgCl_2_, and 0.4 M sucrose and homogenized for 30 s in a blender at maximal speed. The homogenate was filtered through surgical gauze, and the filtrate was centrifuged at 4 °C for 1 min at 500*g*; the supernatant was further centrifuged for 10 min at 1,500*g*. Pelleted chloroplasts were osmotically swollen by resuspension in a sucrose-lacking buffer. The suspension was immediately diluted 1:1 with sucrose-containing buffer, kept on ice in the dark, and used within a few hours from the preparation. Chlorophyll content was calculated after diluting with 80% (v/v) acetone on the basis of Arnon’s formula. The basal rate of photosynthetic electron transport was measured by following light-driven ferricyanide reduction. Membrane aliquots corresponding to 3 μg of chlorophyll were incubated at 24 °C in wells of 96-well-plates in a final volume of 200 μL in the presence of 20 mM Tricine-NaOH buffer (pH 8.0), 10 mM NaCl, 5 mM MgCl_2_, 0.2 M sucrose, and 2 mM K_3_Fe(CN)_6_. The assay was initiated by exposure to saturating light (600 μmol m^–2^ s^–1^), and the rate of ferricyanide reduction was measured at 30 s intervals for 10 min using a Ledetect 96 plate reader (Labexim, Lengau, Austria) equipped with a LED plugin at 420 nm. Activity was calculated over the linear portion of the curve from a molar extinction coefficient of 1,000 M^–1^ cm^–1^. Rubrolides were dissolved in DMSO to obtain 10 mM solutions, which were then diluted with H_2_O, as appropriate. Their effect upon the Hill reaction was evaluated in parallel assays in which the compounds were added to the reaction mixture to concentrations ranging from 0.05 to 100 μM. Each dose was carried out at least in triplicate, and results were expressed as percentage of untreated controls. Mean values ± SE over replicates are reported. The concentrations causing 50% inhibition (IC_50_) and their confidence limits were estimated by nonlinear regression analysis using Prism 6 for Windows, version 6.03 (GraphPad Software).

Phosphorylating electron flow was evaluated under the same conditions but in the presence of 0.5 mM ADP and 2 mM K_2_HPO_4_. Uncoupled activity was measured following the addition of 2 mM NH_4_Cl to the basal reaction mixture containing aliquots of membrane preparations corresponding to 1.5 μg of chlorophyll. To evaluate the effect on PSII only, uncoupled activity was blocked by the addition of the cytochrome b6f inhibitor 2,5-dibromo-6-isopropyl-3-methyl-1,4-benzoquinone (DBMIB) at 2 mM, and an electronic transfer from H_2_O to ferricyanide that excludes PSI (H_2_O → PSII → D1 protein → phenylendiamine → ferricyanide) was restored through the addition of 0.1 mM phenylenediamine. In this case, due to the lower reduction rate, aliquots of membrane preparations corresponding to 6 μg of chlorophyll were used.

### Computational Studies (Molecular Docking)

The structure of the PSII-LHCII supercomplex was prepared for molecular docking by removing H_2_O molecules, ions, lipids, and other solvent molecules. Then, polar hydrogens were added, assigning Gasteiger charges and fusing nonpolar hydrogen atoms. The macromolecule template was constructed from a structure obtained from electron microscopy with reconstruction method of single particle (PDB code: 3JCU).^21^ AutoGrid was used to generate grid maps. The grids were designed including the previously found active site (positioning of amino acids). The box dimensions were defined with size 11.5 × 11.5 × 11.5 Å, and the grid spacing was set to 0.375 Å. All the molecules were previously optimized with the AMBER force field, and **2**, **4**, **10**, **20**, and the control diuron were taken as reference structures. For the molecular docking calculation, AutoDoc vina^22^ was used, which employs Monte-Carlo iterated local search method. Orientations with lower energy, higher populations, and presenting hydrogen bonds were considered for the analysis of the most active molecules. To verify the interactions obtained, a comparative docking was performed with SwiisDock,^23^ with the same box size and locations. After docking, the poses and interactions were analyzed using Chimera UCSF.^27^

## Supporting Information Available

Characterization data of newly synthesized compounds and copies of the ^1^H and ^13^C NMR spectra, Lipinski data, and docking studies data (PDF)
