# Supplementary material for: Natural Rubrolides and Their Synthetic Congeners as Inhibitors of the Photosynthetic Electron Transport Chain
Source: J Nat Prod. 2024 Sep 6;87(9):2272–80. doi: 10.1021/acs.jnatprod.4c00714 (PMC11443480; doi:10.1021/acs.jnatprod.4c00714)
Supplement: Supplementary file 1 — np4c00714_si_001.pdf [file np4c00714_si_001.pdf]

# SUPPORTING INFORMATION

# Natural Rubrolides and Their Synthetic Congeners as Inhibitors of the Photosynthetic Electron Transport Chain

Milandip Karak,[‡] Jaime A. M. Acosta,[‡] Héctor F. Cortez-Hernandez,[§] Johnny L. Cardona,[§] Giuseppe Forlani[¶]* and Luiz C. A. Barbosa[‡]*

[‡]Department of Chemistry, Universidade Federal de Minas Gerais, Av. Pres. Antônio Carlos, 6627, Campus Pampulha, CEP 31270-901, Belo Horizonte, MG, Brazil.

[§]School of Chemical Technology, Faculty of Technology, Universidad Tecnológica de Pereira, Carrera 27 #10-02, Barrio Álamos, Código postal: 660003, Pereira, Risaralda, Colombia.

[¶]Department of Life Science and Biotechnology, Università di Ferrara, via L. Borsari 46, I-44121 Ferrara, Italy.

***Corresponding Authors**: Luiz C. A. Barbosa: lcab@ufmg.br
Giuseppe Forlani: flg@unife.it

## Table of Contents


| | |
|---|---|
| 1. Characteristic data for 4-aryl-3-chlorobutenolides (**11a-f**) | S3 |
| 2. Characteristic data for the rubrolide analogous (**12–20**) | S4 |
| 3. NMR spectra of new rubrolide intermediates and analogous | S9 |
| 4. Physicochemical properties, lipophilicity and water solubility | S22 |
| 5. Molecular docking results | S22 |
| 6. References | S24 |

## 1. Characteristic data for 4-aryl-3-chlorobutenolides (11a-f)

Physical and spectroscopic data for the compound **11a** was in excellent agreement with that reported in the literature.[1] Characteristic data and copy of NMR spectra for compound **11e** had been previously documented in our research.[2]

*3-chloro-4-(5-chloro-2-methoxyphenyl)furan-2(5H)-one (**11b**)*

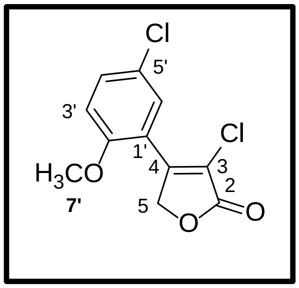


Yield: 85%; physical state and color: white solid; eluent (EtOAc/hexane (v/v)): 2:8; Mp: 205.1-206.3 °C; FTIR ($cm^{-1}$): $\bar{\upsilon}_{max}$ 3028, 2982, 2851, 1750, 1624, 1496, 1339, 1259, 1184, 824, 756, 732; $^{1}$H NMR (400 MHz, $CDCl_3$) $\delta$: 7.85 (s, 1H, H-6'), 7.42 (d, *J* = 8.4 Hz, 1H, H-3'), 6.96 (d, *J* = 8.4 Hz, 1H, H-4') 5.28 (s, 2H, H-5) and 3.89 (s, 3H, -$OCH_3$); $^{13}$C NMR (100 MHz, $CDCl_3$) $\delta$: 168.7 (C-2), 156.1 (C-2'), 151.3 (C-4), 132.2 (C-4'), 129.6 (C-6'), 126.1 (C-1'), 119.3 (C-5'), 119.0 (C-3), 112.9 (C-3'), 72.0 (C-5) and 56.0 ($OCH_3$); HRMS (ESI) $[M+H]^+$ calculated for $C_{11}H_9Cl_2O_3$, 258.9929; found, 258.9907.

*3-chloro-4-(4-fluorophenyl)furan-2(5H)-one (**11c**)*

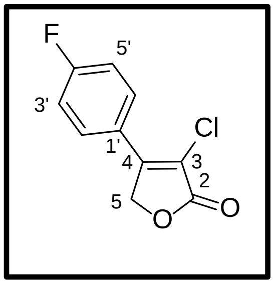


Yield: 78%; physical state and color: light brown solid; eluent (EtOAc/hexane (v/v)): 2:8; Mp: 120.1-121.7 °C; FTIR ($cm^{-1}$): $\bar{\upsilon}_{max}$ 3037, 2916, 1753, 1607, 1506, 1437, 1259, 1188, 998; $^{1}$H NMR (400 MHz, $CDCl_3$): $\delta$ : 7.93-7.88 (m, 2H, H-2' and H-6'), 7.23 (t, *J* = 8.2 Hz, 2H, H-3' and H-5'), and 5.23 (s, 2H, H-5); $^{13}$C NMR (100 MHz, $CDCl_3$) $\delta$: 168.9 (C-2), 164.4 (d, *J* = 254.65 Hz, C-4'), 150.7 (C-4), 129.6 (d, *J* = 8.8 Hz, 2C, C-2' and C-6'), 125.1 (d, *J* = 3.2 Hz, C-1'), 117.1 (C-3), 116.6 (d, *J* = 22.0 Hz, 2C, C-3' and C-5'), 70.0 (C-5); HRMS (ESI) $[M+H]^+$ calculated for $C_{10}H_7ClFO_2$, 213.0119; found, 213.0112.

*3-chloro-4-(4-fluoro-2-methoxyphenyl)furan-2(5H)-one (**11d**)*

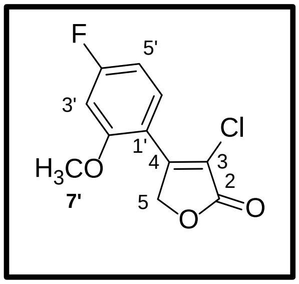


Yield: 62%; physical state and color: white solid; eluent (EtOAc/hexane (v/v)): 8:2; $^{1}$H NMR (400 MHz, $CDCl_3$): $\delta$: 8.05-7.87 (br, 1H, H-6'), 7.42 (m, 6.88-6.68, 2H, H-3' and H-5'), 5.29 (s, 2H, H-5) and 3.89 (s, 3H, -$OCH_3$); $^{13}$C NMR (100 MHz, $CDCl_3$): $\delta$: 169.0 (C-2), 165.3 (d, *J* = 253.3 Hz, C-4'), 159.3 (C-2'), 151.8 (C-4), 131.7 (C-6'), 117.6 (C-3), 114.2 (C-

1'), 108.0 (d, $J$ = 21.9 Hz, C-5'), 100.0 (d, $J$ = 25.9 Hz, C-3'), 72.1 (C-5) and 56.0 ($OCH_3$).; HRMS (ESI) $[M+H]^+$ calculated for $C_{11}H_9ClFO_3$, 243.0224; found, 243.0231.

3-*chloro-4-(3-chloro-4-methoxyphenyl)furan-2(5H)-one* (***11f***)

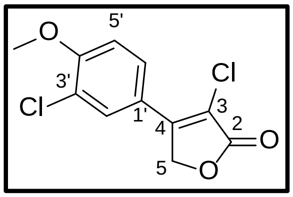


Yield: 51%; physical state and color: white solid; eluent (EtOAc/hexane (v/v)): 17:3; Mp: 160.8-161.7 ºC. FTIR ($cm^{-1}$): $\bar{\upsilon}_{max}$ 3101, 2950, 1756, 1614, 1598, 1508, 1444, 1329, 1263, 1199, 1075, 1036, 1007, 830, 702; $^1H$ NMR (400 MHz, $CDCl_3$) $\delta$ : 7.85 (s, 1H, H-2′), 7.77 (d, $J$= 7.8 Hz, 1H, H-6′), 7.07 (d, $J$= 7.8 Hz, 1H, H-5′), 5.19 (s, 2H, H-5) and 4.00 (s, 3H, -$OCH_3$). $^{13}C$ NMR (100 MHz, $CDCl_3$) $\delta$ : 169.0 (C-2), 157.6(C-4′), 150.0 (C-4), 129.1 (C-2′), 127.4 (C-6′), 123.7 (C-1′), 122.0 (C-3′), 116.7 (C-3), 112.2 (C-5′), 69.8 (C-5) and 56.4 (-$OCH_3$). HRMS (ESI-TOF) *m/z* $[M+H]^+$ calculated for $C_{11}H_9Cl_2O_3$, 258.9923; found, 258.9928.

## 2. Characteristic data for the rubrolide analogous (12–20)

Our research group has previously documented comprehensive experimental and characterization details of natural rubrolides B, E, F, 3"-bromo rubrolide F, I, K, and O (compounds **1–7**), along with intermediate compounds **8–10**. These details encompassed their synthetic procedures, physical characteristics, and spectroscopic data which can be found under the following references.[2-3]

*(Z)-3-chloro-5-(4-methoxybenzylidene)-4-phenylfuran-2(5H)-one* (***12***)

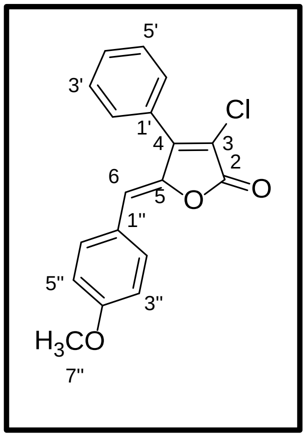


Yield: 41%; physical state and color: yellow solid; eluent (EtOAc/hexane (v/v)): 2:8; Mp: 178.5-179.7 °C; FTIR ($cm^{-1}$): $\bar{\upsilon}_{max}$ 2999, 2846, 2015, 1760, 1645, 1596, 1507, 1442, 1306, 1252, 1004, 627; $^1H$ NMR (400 MHz, $CDCl_3$) $\delta$: 7.72 (d, $J$ = 8.7 Hz, 2H, H-2'' and H-6''), 7.59–7.49 (m, 5H, H-2' to H-6'), 6.91 (d, $J$ = 8.7 Hz, 2H, H-3'' and H-5''), 6.09 (s, 1H, H-6), 3.84 (s, 3H, -$OCH_3$); $^{13}C$ NMR (100 MHz, $CDCl_3$) $\delta$: 160.9 (C-2), 150.2 (C-4''), 147.0 (C-4), 144.9 (C-5), 132.8 (2C, C-2' and C-6'), 130.1 (C-1'), 129.2 (2C, C-2'' and C-6''), 129.1 (2C, C-3' and C-5'), 128.4 (C-4'), 125.6 (C-1''), 117.6 (C-3), 114.9 (C-6), 114.6 (2C, C-3'' and C-5''), 55.5 (-$OCH_3$); HRMS (ESI-TOF) *m/z* $[M+H]^+$ calculated for $C_{18}H_{14}ClO_3$, 313.0631; found, 313.0625.

*(Z)-3-chloro-5-(2-chloro-4-fluorobenzylidene)-4-phenylfuran-2(5H)-one (**13**)*

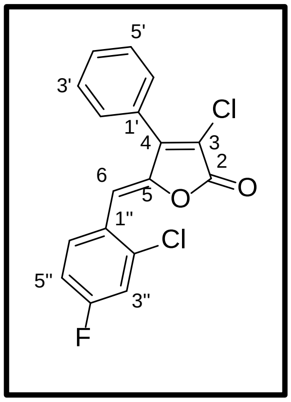


Yield: 43%; physical state and color: yellow solid; eluent (EtOAc/hexane (v/v)): 15:85; Mp: 145.3-147.0 °C; FTIR ($cm^{-1}$): $\bar{\upsilon}_{max}$ 3083, 2929, 2029, 1778, 1596, 1568, 1487, 1248, 1008, 882, 747, 695; $^{1}H$ NMR (400 MHz, $CDCl_3$) $\delta$: 8.26 (dd, *J* = 8.8 Hz, *J* = 6.2 Hz, 1H, H-3''), 7.63–7.55 (br m, 5H, H-2' to H-6'), 7.16 (dd, *J* = 2.5 Hz, 8.3 Hz, 1H, H-6''), 7.08 (td, *J* = 2.5 Hz, 8.8 Hz, 1H, H-5''), 6.60 (s, 1H, H-6); $^{13}C$ NMR (100 MHz, $CDCl_3$) $\delta$: 164.3 (C-2), 162.7 (d, *J* = 255.1 Hz, C-4''), 150.1 (C-5), 147.2 (C-4), 133.5 (d, *J* = 9.1 Hz, C-2''), 130.9 (2C, C-4', C-6''), 129.3 (4C, C-2', C-3', C-5' and C-6'), 127.9 (C-1'), 127.1 (d, *J* = 3.0 Hz, C-1''), 117.4 (d, *J* = 24.9 Hz, C-5''), 115.7 (C-3), 115.1 (d, *J* = 21.2 Hz, C-3''), 109.0 (C-6); HRMS (ESI-TOF) *m/z* $[M+H]^+$: calculated for $C_{17}H_{10}Cl_2FO_2$, 335.0036; found, 335.0038.

*(Z)-5-((2-bromopyridin-4-yl)methylene)-3-chloro-4-(5-chloro-2-methoxyphenyl) furan-2(5H)-one (**14**)*

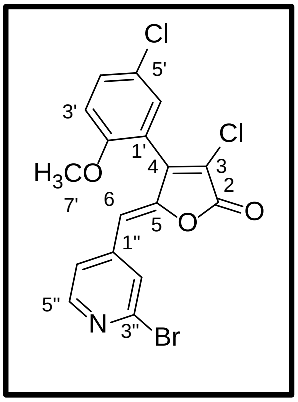


Yield: 12% (this compound was acquired as a mixture of *Z/E* isomers, with the isolated pure Z-isomer yielding 12%); physical state and color: yellow solid; eluent (EtOAc/hexane (v/v)): 1:9; Mp: 188.1-188.8 °C; FTIR ($cm^{-1}$): $\bar{\upsilon}_{max}$ 3064, 2850, 2166, 2035, 1770, 1484, 1408, 1374, 1271, 1178, 1093, 914, 886, 809, 755, 693; $^{1}H$ NMR (400 MHz, $CDCl_3$) $\delta$: 8.43 (s, 1H, H-2''), 8.17 (d, *J* = 8.3 Hz, 1H, H-5''), 7.59 - 7.47 (m, 2H, H-4' and H-6'), 7.02 (d, *J* = 8.9 Hz, 1H, H-3'), 5.83 (s, 1H, H-6), 3.85 (s, 3H, $-CH_3$); $^{13}C$ NMR (100 MHz, $CDCl_3$) $\delta$: 163.6 (C-2), 155.7 (C-2'), 151.6 (C-5''), 148.2 (C-3''), 146.5 (C-4''), 142.6 (C-5), 139.1 (C-6'), 132.2 (C-4'), 130.0 (C-6''), 128.6 (C-2''), 128.3 (C-1''), 128.1 (C-5'), 123.1 (C-3), 117.8 (C-1'), 113.2 (C-3'), 108.3 (C-6), 56.2 ($-OCH_3$); HRMS (ESI-TOF) *m/z* $[M+H]^+$: calculated for $C_{17}H_{11}BrCl_2NO_3$, 425.9294; found, 425.9285.

*(Z)-5-(benzo[d][1,3]dioxol-4-ylmethylene)-3-chloro-4-(5-chloro-2-methoxyphenyl) furan-2(5H)-one (**15**)*

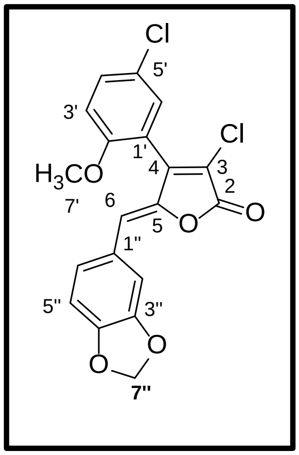


Yield: 38%; physical state and color: yellow solid; eluent (EtOAc/hexane (v/v)): 2:8; Mp: 134.9-136.2 °C; FTIR ($cm^{-1}$): $\bar{\upsilon}_{max}$ 3018, 2898, 2021, 1767, 1482, 1444, 1250, 1003, 877, 601, 624; $^1$H NMR (400 MHz, $CDCl_3$) $\delta$: 7.47 (s, 2H, H-6' and H-2''), 7.26 (br s, 1H, H-4'), 7.13 (d, $J$ = 7.6 Hz, 1H, H-3'), 7.02 (d, $J$ = 8.8 Hz, 1H, H-6''), 6.82 (d, $J$ = 8.8 Hz, 1H, H-5''), 6.02 (s, 2H, H-7''), 5.85 (s, 1H, H-6), 3.86 (s, 3H, $-OCH_3$).; $^{13}$C NMR (100 MHz, $CDCl_3$) $\delta$: 164.4 (C-2), 156.6 (C-2'), 149.0 (C-3''), 148.4 (C-4''), 146.7 (C-5), 144.7 (C-4), 131.6 (C-6'), 130.0 (C-4'), 126.9 (C-1''), 126.6 (C-6''), 125.7 (C-1'), 120.4 (C-5'), 118.4 (C-3), 113.9 (C-3'), 113.0 (C-6), 110.1 (C-5''), 108.6 (C-2''), 101.6 (C-7''), 56.1 ($-OCH_3$).; HRMS (ESI-TOF) *m/z* $[M+H]^+$: calculated for $C_{19}H_{13}Cl_2O_5$, 391.0140; found, 391.0225.

*(Z)-3-chloro-4-(4-fluorophenyl)-5-((5-phenylthiophen-2-yl)methylene)furan-2(5H)-one (**16**)*

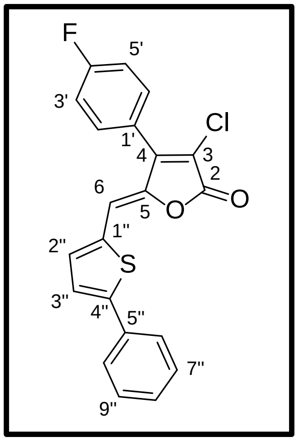


Yield: 50%; physical state and color: orange solid; eluent (EtOAc/hexane (v/v)): 15:85; Mp: 218.3-219.8 °C (decomp.) FTIR ($cm^{-1}$): $\bar{\upsilon}_{max}$ 3066, 2922, 2854, 2011, 1760, 1632, 1596, 1501, 1440, 1222, 1161, 1005, 843, 796, 762, 687, 499; $^1$H NMR (400 MHz, $(CD_3)_2SO$) $\delta$: 7.74 (d, $J$ = 7.5 Hz, 2H, H-5'' and H-9''), 7.69 (dd, $J$ = 8.1 Hz, 5.6 Hz, 2H, H-2' and H-6'), 7.74 (dd, $J$ = 8.7 Hz, 3.6 Hz, 2H, H-6'' and H-8''), 7.52 – 7.42 (m, 4H, H-3'', H-7'', H-3' and H-5'), 7.41 – 7.34 (m, 1H, H-2''), 6.75 (s, 1H, H-6); $^{13}$C NMR (100 MHz, $(CD_3)_2SO$) $\delta$: 163.1 (C-2), 160.6 (d, $J$ = 285.3 Hz, C-4′), 149.3 (C-5), 147.9 (C-4''), 143.5 (C-4), 134.9 (C-1''), 134.8 (5''), 133.0 (C-2''), 131.6 (d, $J$ = 8.9 Hz, 2C, C-2′ and C-6′), 129.2 (2C, C7'' and C9''), 128.6 (C-8'), 125.5 (2C, C6'' and C10''), 124.5 (C-1'), 123.7 (C-3), 117.1 (C-3''), 116.3 (d, $J$ = 22.1 Hz, 2C, C-3′ and C-5′), 108.6 (C-6); HRMS (ESI-TOF) *m/z* $[M+H]^+$: calculated for $C_{21}H_{12}ClFO_2S$, 383.0303; found, 383.0311.

*(E/Z)-5-((2-bromopyridin-4-yl)methylene)-3-chloro-4-(4-fluorophenyl)furan-2(5H)-one (**17**)*

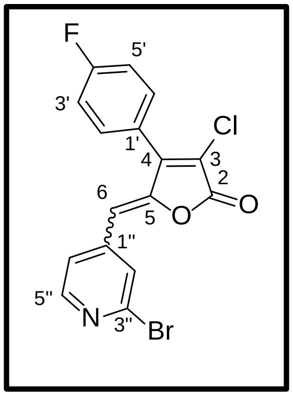


Yield: 34%; *Z/E* ratio (0.74/0.26); physical state and color: yellow solid; eluent (EtOAc/hexane (v/v)): 35:65; Mp: 150.3-152.8 °C; FTIR ($cm^{-1}$): $\bar{\upsilon}_{max}$ 3101, 2929, 2032, 1757, 1599, 1570, 1503, 1375, 1233, 1159, 1089, 1017, 834, 752, 679; $^{1}H$ NMR (400 MHz, $CDCl_3$) $\delta$: 8.47 (br s, 0.74H/0.26H, H-5’’), 8.25 (d, *J* = 7.8 Hz, 0.26H, H-6’’), 8.22 (d, *J* = 8.2 Hz, 0.74H, H-6’’), 7.65-7.47 (m, 0.74H, H-2’’, H-2’ and H-6’; 0.26H, H-2’’), 7.46-7.39 (m, 0.26H, H-2’ and H-6’), 7.38-7.16 (m, 0.74H, H-3’ and H-5’), 7.08-6.92 (m, 0.26H, H-3’ and H-5’), 6.05 (s, 0.74H, H-6), 5.89 (s, 0.26H, H-6); $^{13}C$ NMR (100 MHz, $CDCl_3$) $\delta$: 167.6 (0.74C, C-2), 167.4 (0.26C, C-2), 164.3 (d, *J* = 231.9 Hz, 0.74C, C-4’), 164.5 (d, *J* = 234.7 Hz, 0.26C, C-4’), 151.5 (0.74C, C-5’’), 151.3 (0.26C, C-5’’), 148.5 (0.74C, C-3’’), 148.3 (0.74C, C-5), 142.7 (0.74C, C-4), 142.2 (0.26C, C-4), 139.0 (0.74C, C-1’’), 138.9 (0.26C, C-1’’), 131.2 (d, *J* = 8.6 Hz, 0.74C, C-2’ and C-6’), 131.1 (d, *J* = 8.1 Hz, 0.26C, C-2’ and C-6’), 128.6 (0.26C, C-6’’), 128.5 (0.74C, C-2’’), 128.4 (0.26C, C-2’’), 128.0 (0.74C, C-6’’), 123.5 (d, *J* = 3.2 Hz, C-1’’), 120.6 (C-3), 116.7 (d, *J* = 22.1 Hz, 0.74C, C-3’ and C-5’), 115.8 (d, *J* = 21.8 Hz, 0.26C, C-3’ and C-5’), 108.9 (0.74C, C-6), and 107.7 (0.26C, C-6); HRMS (ESI) *m/z* $[M+H]^+$: calculated for $C_{16}H_9BrClFNO_2$, 379.9489; found, 379.9457.

*(Z)-5-(benzo[d][1,3]dioxol-4-ylmethylene)-3-chloro-4-(4-fluoro-2-methoxyphenyl) furan-2(5H)-one (**18**)*

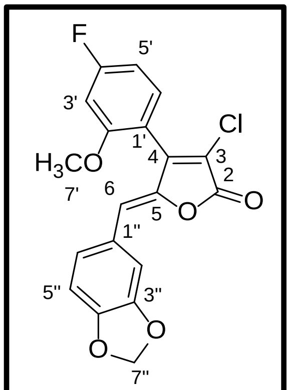


Yield: 57%; physical state and color: yellow solid; eluent (EtOAc/hexane (v/v)): 2:8; Mp: 141.8-142.6 °C; FTIR ($cm^{-1}$): $\bar{\upsilon}_{max}$ 3091, 2912, 2038, 1756, 1610, 1594, 1494, 1448, 1271, 1187, 1105, 1021, 959, 925, 872, 791, 677, 614; $^{1}H$ NMR (400 MHz, $CDCl_3$) $\delta$: 7.45 (s, 1H, H-6’), 7.31-7.21 (m, 1H, H-3’), 7.15-7.05 (m, 1H, H-5’), 6.91-6.72 (m, 3H, H-2’’, H-5’’ and H-6’’), 6.01 (s, 2H, H-7’’), 5.84 (s, 1H, H-6) and 3.86 (s, 3H, -$OCH_3$); $^{13}C$ NMR (100 MHz, $CDCl_3$) $\delta$: 167.0 (d, *J* = 243.1 Hz, C-4’), 164.5 (C-2), 158.5 (d, *J* = 10.0 Hz, C-2’), 148.9 (C-3’’), 148.4 (C-4’’), 147.4 (C-5), 145.1 (C-4), 131.6 (d, *J* = 10.6 Hz, C-6’), 127.0 (C-6’’), 126.5 (C-1’’), 120.0 (C-3), 113.8 (C-6), 112.9 (d, *J* = 3.1 Hz, C-1’), 110.1 (C-2’’), 108.6 (C-5’’), 107.6 (d, *J* = 22.0 Hz, C-5’),

101.6 (C-7’’), 100.2 (d, $J$ = 26.0 Hz, C-3’), and 56.0 (-$OCH_3$). HRMS (ESI-TOF) m/z $[M+H]^+$: calculated for $C_{19}H_{13}ClFO_5$, 375.0436; found, 375.0432.

*(Z)-5-(3-bromo-4-methoxybenzylidene)-3-chloro-4-(3-chloro-4-methoxyphenyl) furan-2(5H)-one **(19)***

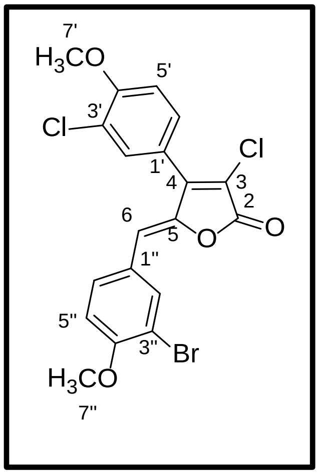


Yield: 70%; physical state and color: yellow solid; eluent (EtOAc/hexane (v/v)): 2:8; Mp: 217.1-219.0 °C; FTIR ($cm^{-1}$): $\bar{\upsilon}_{max}$ 2843, 2008, 1780, 1641, 1593, 1496, 1287, 1270, 1233, 1161, 1065, 1051, 1006, 882, 817, 710, 646; $^1H$ NMR (400 MHz, $CDCl_3$) $\delta$: 7.95 (d, $J$ = 1.9 Hz, 1H, H-2’), 7.80 (dd, $J$ = 1.9 Hz, 8.7 Hz, 1H, H-6’), 7.58 (d, $J$ = 2.0 Hz, 1H, H-2’’), 7.45 (dd, J = 2.0 Hz, 8.5 Hz, 1H, H-6’’), 7.13 (d, $J$ = 8.5 Hz, 1H, H-5’’), 6.94 (d, $J$ = 8.7 Hz, 1H, H-5’), 6.03 (s, 1H, H-6), 4.03 (s, 3H, -$OCH_3$) and 3.96 (s, 3H, -$OCH_3$).; $^{13}C$ NMR (100 MHz, $CDCl_3$) $\delta$: 164.2 (C-2), 156.9 (C-4’’), 156.8 (C-4’), 148.2 (C-4), 145.4 (C-5), 135.6 (C-2’’), 131.5 (C-2’), 130.8 (C-6’), 129.0 (C-6’’), 126.6 (C-1’), 123.6 (C-1’’), 120.9 (C-3’), 118.2 (C-3), 112.9 (C-6), 112.3 (C-5’’), 112.2 (C-3’’), 112.0 (C-5’), 56.4 (2C, -$OCH_3$).; HRMS (ESI-TOF) *m/z* $[M+H]^+$: calculated for $C_{19}H_{14}BrCl_2O_4$, 454.9453; found, 454.9432.

*(Z)-5-(3-bromo-4-hydroxybenzylidene)-3-chloro-4-(3-chloro-4-hydroxyphenyl) furan-2(5H)-one **(20)***

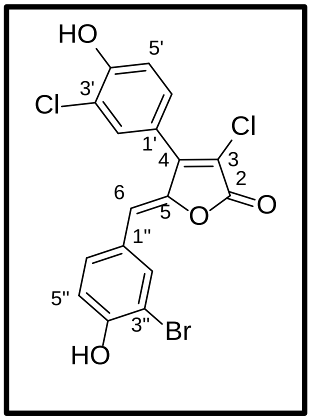


Yield: 92%; physical state and color: yellow solid; eluent (EtOAc/hexane (v/v)): 3:7; Mp: 210-213 °C (decomp.); FTIR ($cm^{-1}$): $\bar{\upsilon}_{max}$ 3347, 2929, 2368, 1750, 1644, 1598, 1497, 1408, 1327, 1278, 1192, 1139, 1026, 911, 826, 808, 668; $^1H$ NMR (400 MHz, $CDCl_3$) $\delta$: 9.52 (br, 2H, -OH), 8.03 (d, $J$ = 1.6 Hz, 1H, H-2′), 7.72 (dd, $J$ = 1.6 Hz, 8.5 Hz, 1H, H-6’), 7.63 (d, $J$ = 1.9 Hz, 1H, H-2’’), 7.45 (dd, $J$ = 1.9 Hz, 8.4 Hz, 1H, H-6’’), 7.23 (d, $J$ = 8.4 Hz, 1H, H-5’’), 7.07 (d, $J$ = 8.5 Hz, 1H, H-5’), and 6.29 (s, 1H, H-6); $^{13}C$ NMR (100 MHz, $CDCl_3$) $\delta$: 164.5 (C-2), 156.1 (C-4’’), 155.8 (C-4’), 149.6 (C-4), 146.1 (C-5), 136.4 (C-2’’), 132.5 (C-2’), 131.7 (C-6’), 130.3 (C-6’’), 127.3 (C-1’), 121.8 (C-1’’), 121.2 (C-3’), 118.0 (C-5’’), 118.0 (C-3), 117.6 (C-

5’), 113.6 (C-6), 110.9 (C-3’’); HRMS (ESI-TOF) *m/z* [M+H]$^+$: calculated for $C_{17}H_{10}BrCl_2O_4$, 426.9140; found, 426.9132.

### 3. NMR spectra of the new rubrolide intermediates and analogous

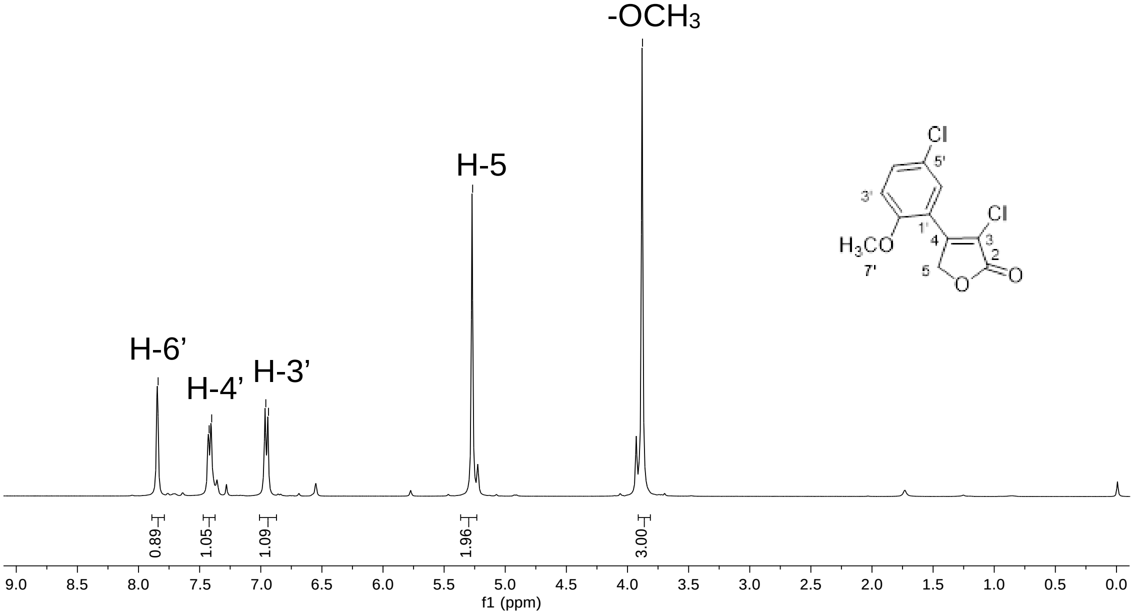


**Fig. S1** $^1$H NMR (400 MHz, $CDCl_3$) of compound **11b**.

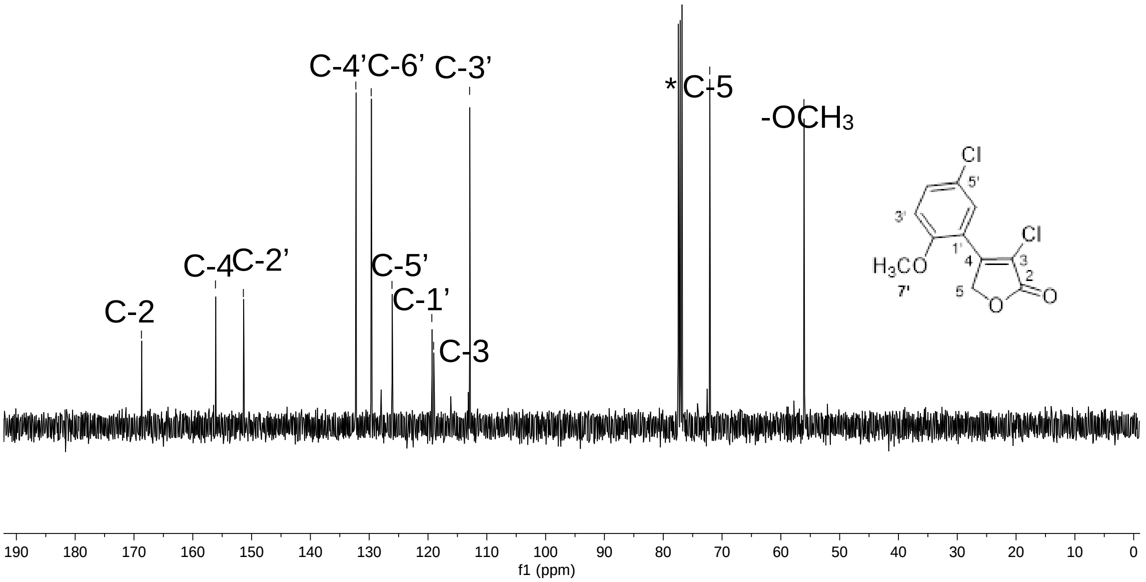


**Fig. S2** $^{13}$C NMR (100 MHz, $CDCl_3$) of compound **11b**. *Solvent residues signals.

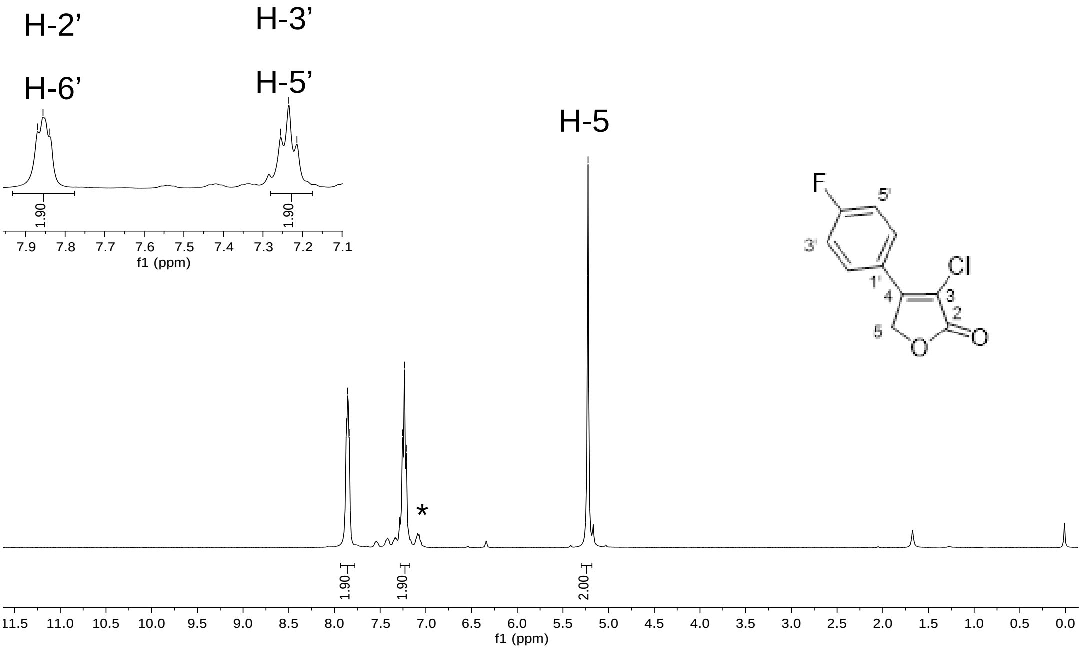


**Fig. S3** $^{1}$H NMR (400 MHz, $CDCl_3$) of compound **11c**. *Solvent residues and $H_2O$ signals.

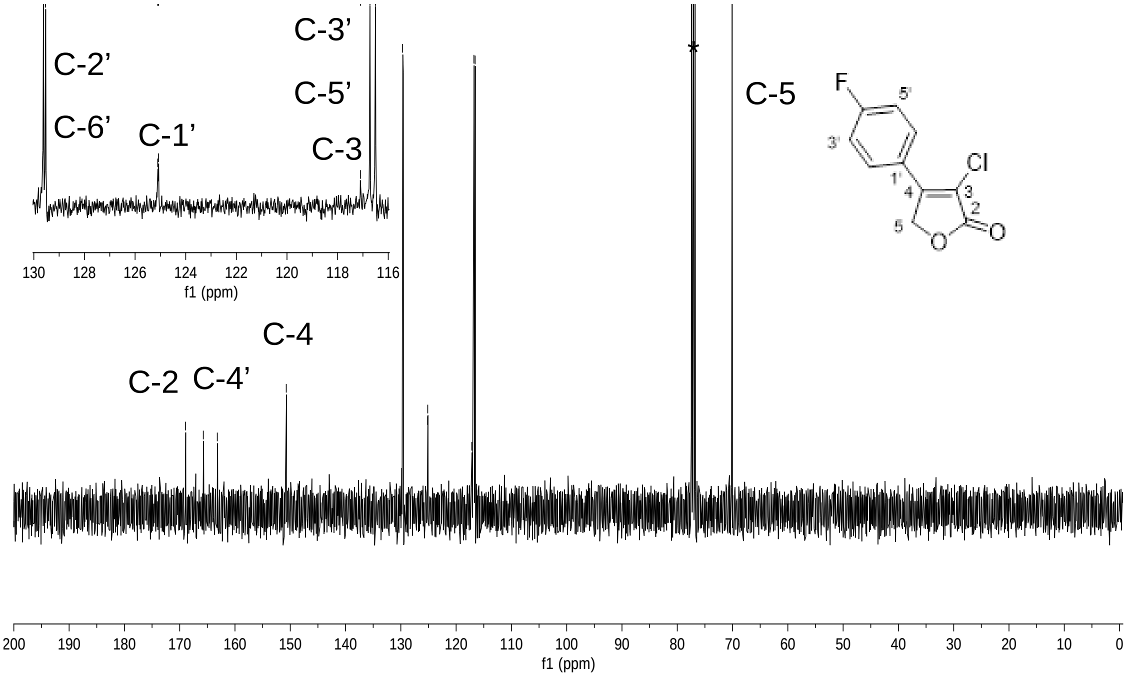


**Fig. S4** $^{13}$C NMR (100 MHz, $CDCl_3$) of compound **11c**. *Solvent residues signals.

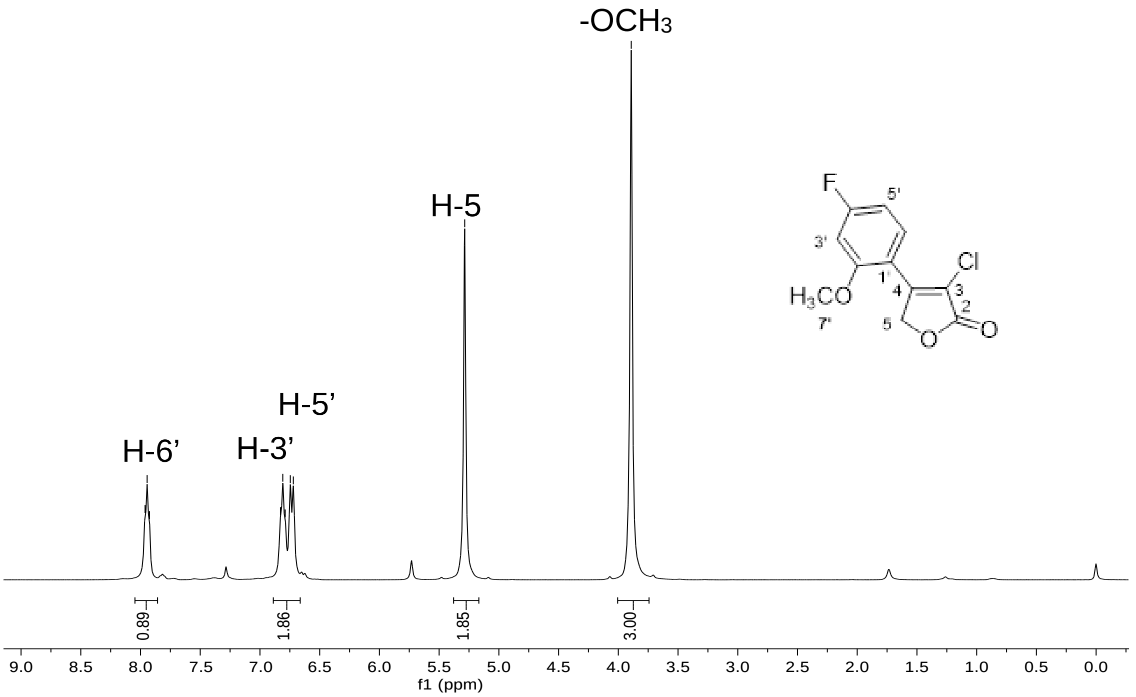


**Fig. S5** $^{1}H$ NMR (400 MHz, $CDCl_3$) of compound **11d**.

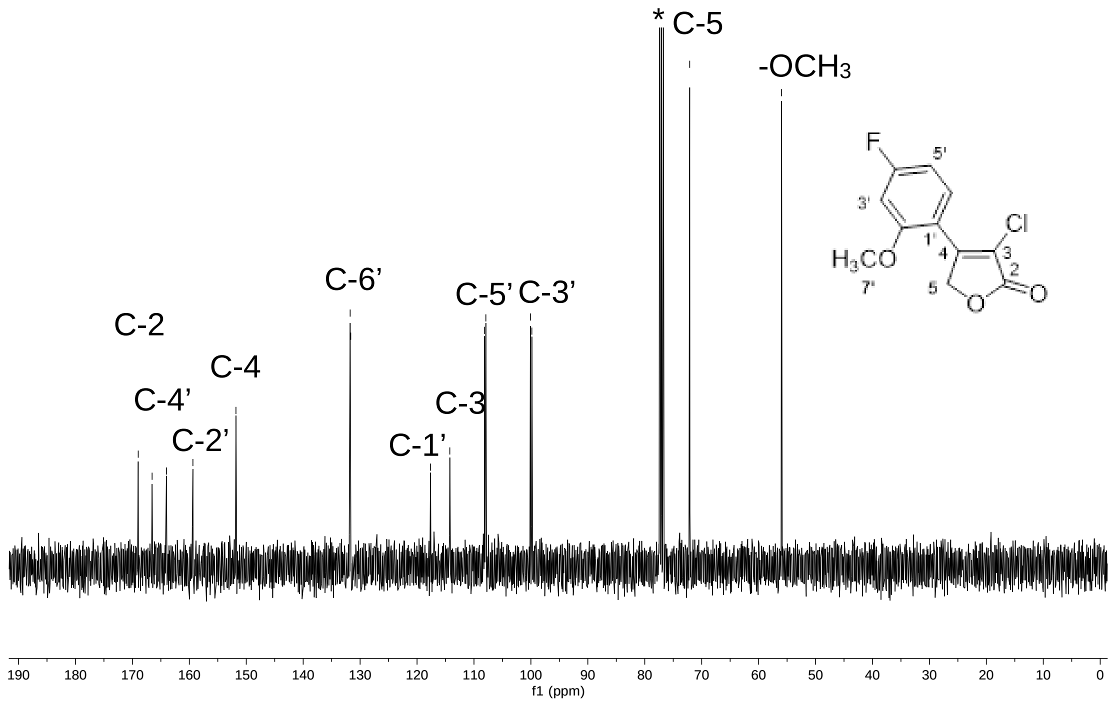


**Fig. S6** $^{13}C$ NMR (100 MHz, $CDCl_3$) of compound **11d**. *Solvent residues signals.

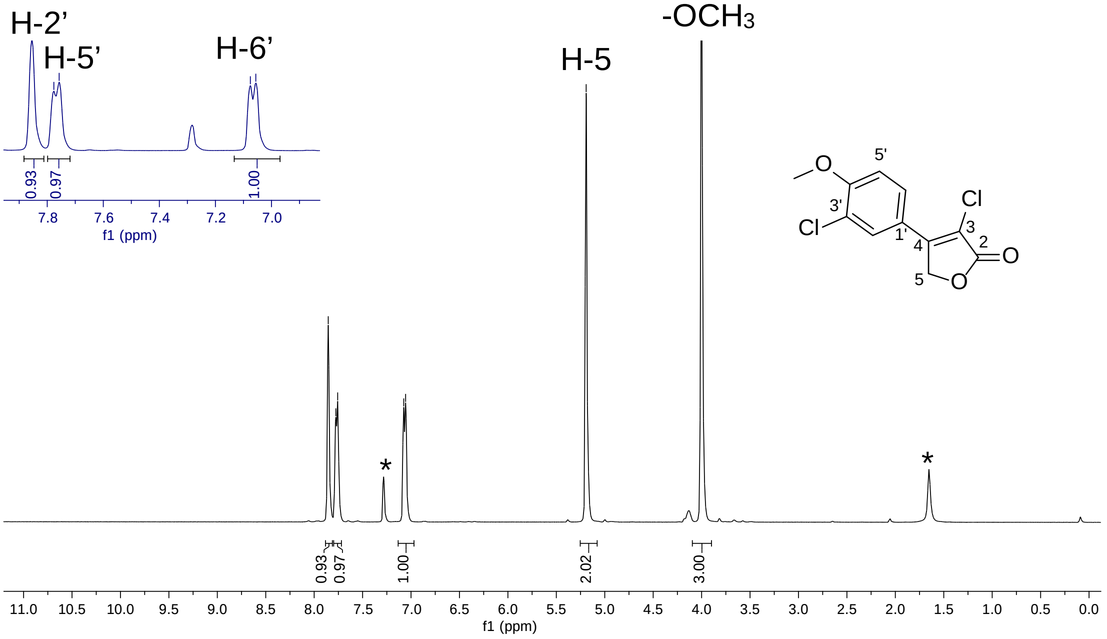


**Fig. S7** $^{1}H$ NMR (400 MHz, $CDCl_3$) of compound **11f**. *Solvent residues signals.

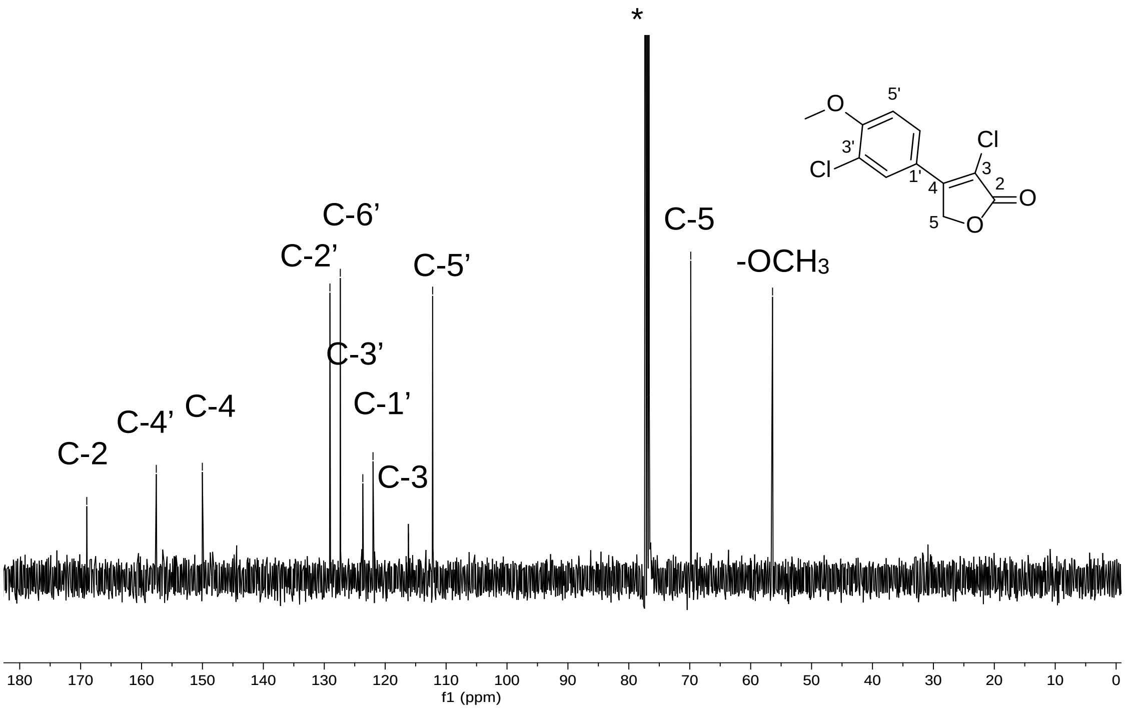


**Fig. S8** $^{13}C$ NMR (100 MHz, $CDCl_3$) of compound **11f**. *Solvent residues signals.

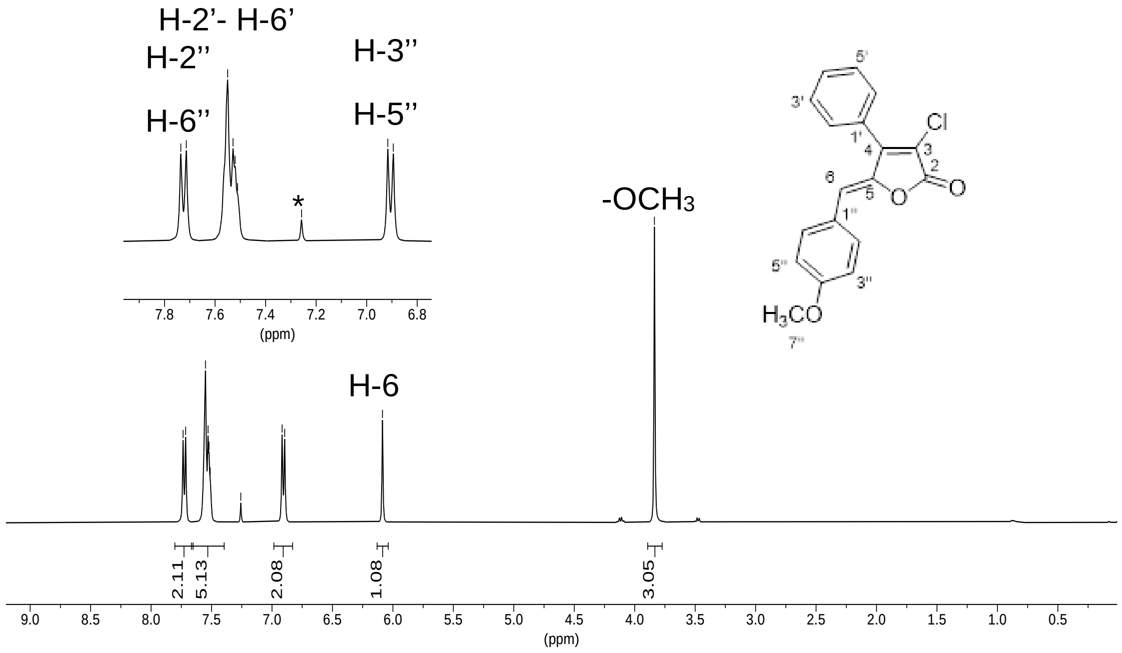


**Fig. S9** ${}^{1}H$ NMR (400 MHz, $CDCl_3$) of compound **12**. *Solvent residues and $H_2O$ signals.

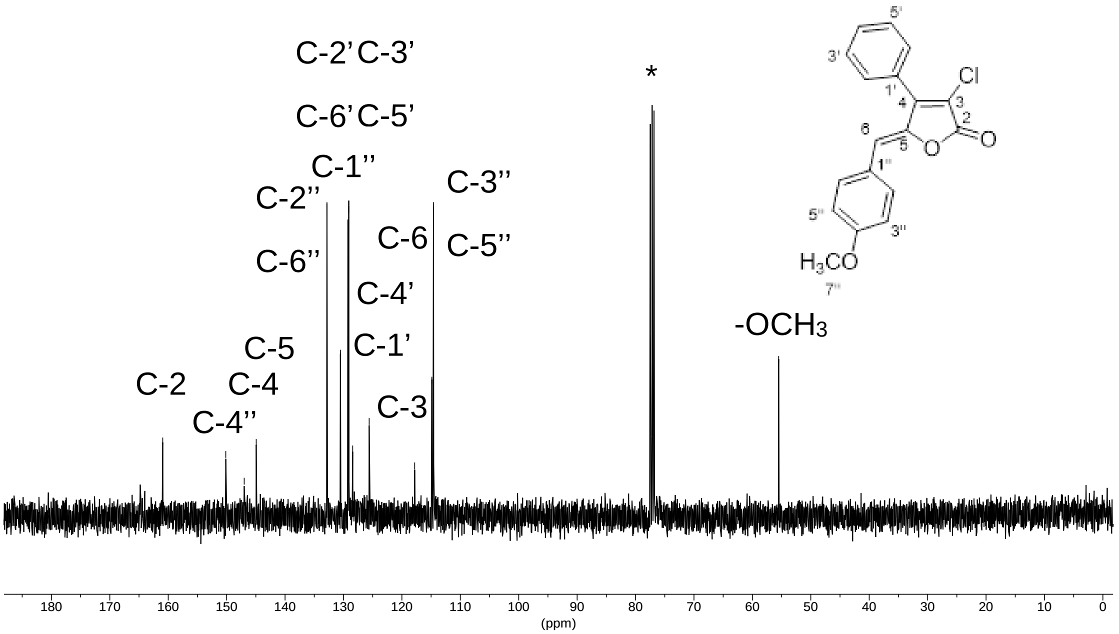


**Fig. S10** ${}^{13}C$ NMR (100 MHz, $CDCl_3$) of compound **12**. *Solvent residues signals.

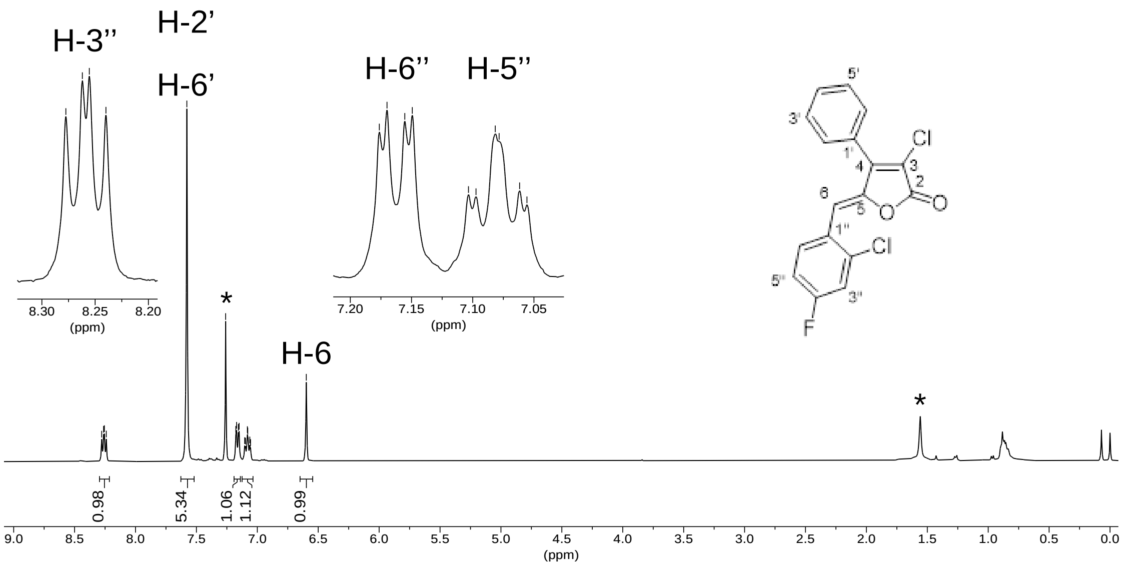


**Fig. S11** $^{1}H$ NMR (400 MHz, $CDCl_3$) of compound **13**. *Solvent residues and $H_2O$ signals.

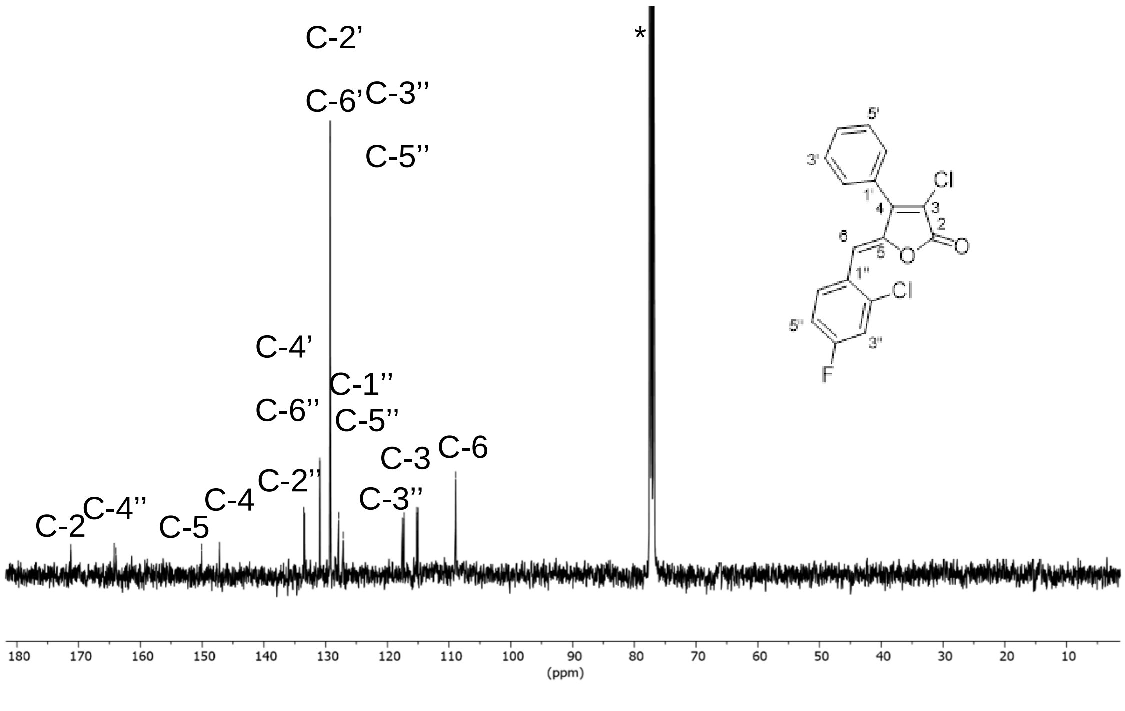


**Fig. S12** $^{13}C$ NMR (100 MHz, $CDCl_3$) of compound **13**. *Solvent residues signals.

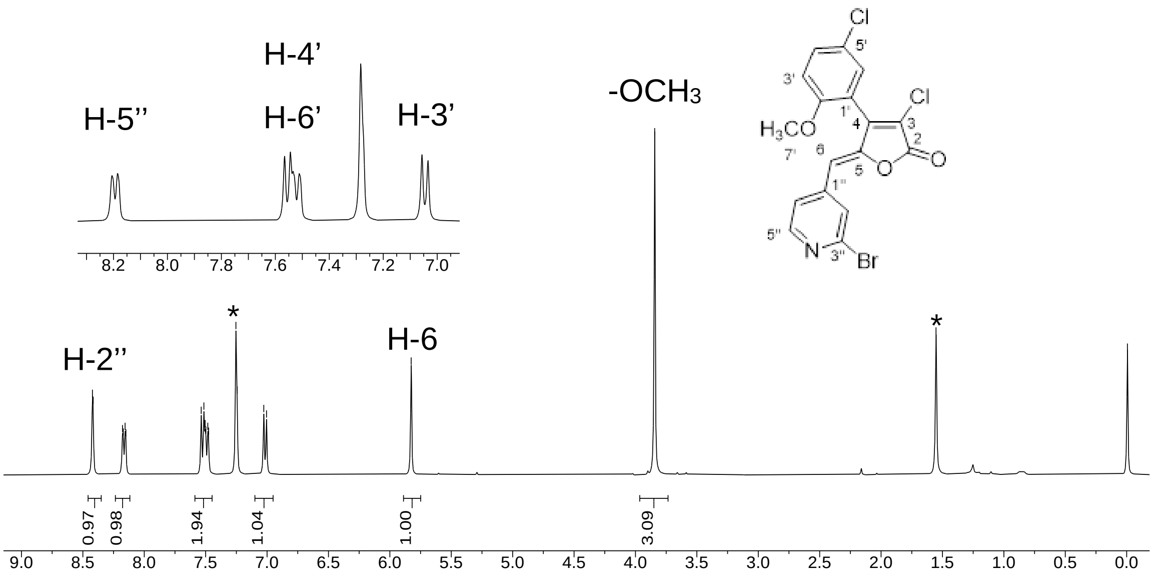


**Fig. S13** $^{1}H$ NMR (400 MHz, $CDCl_3$) of compound **14**. *Solvent residues and $H_2O$ signals.

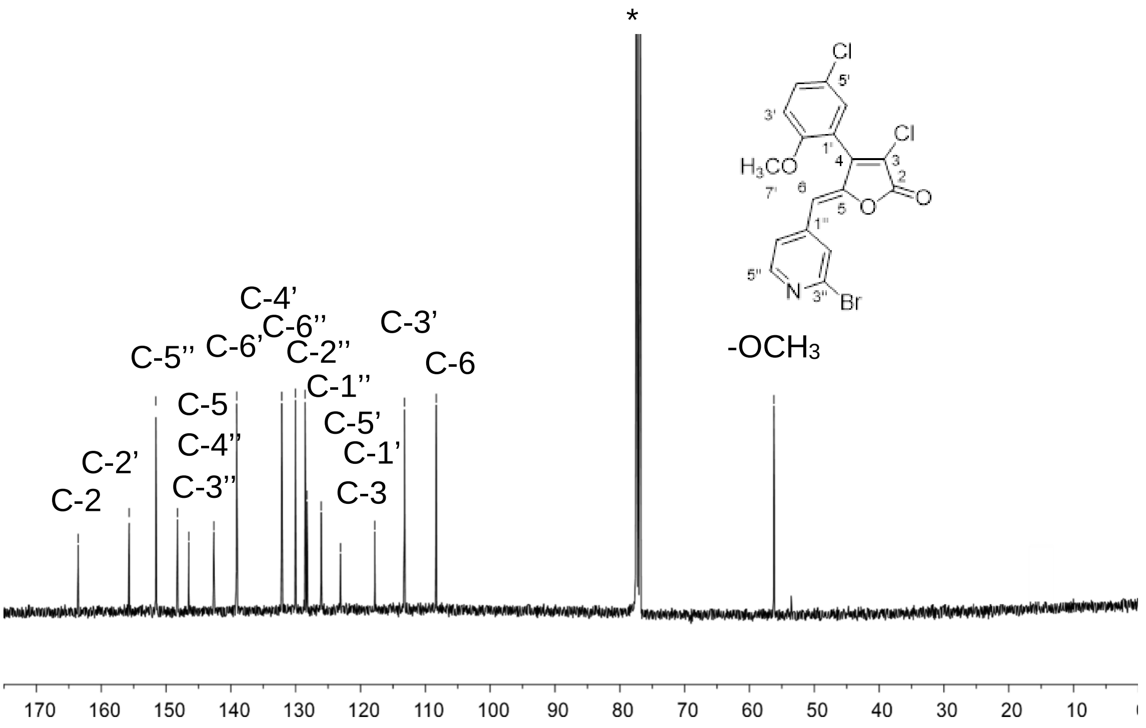


**Fig. S14** $^{13}C$ NMR (100 MHz, $CDCl_3$) of compound **14**. *Solvent residues signals.

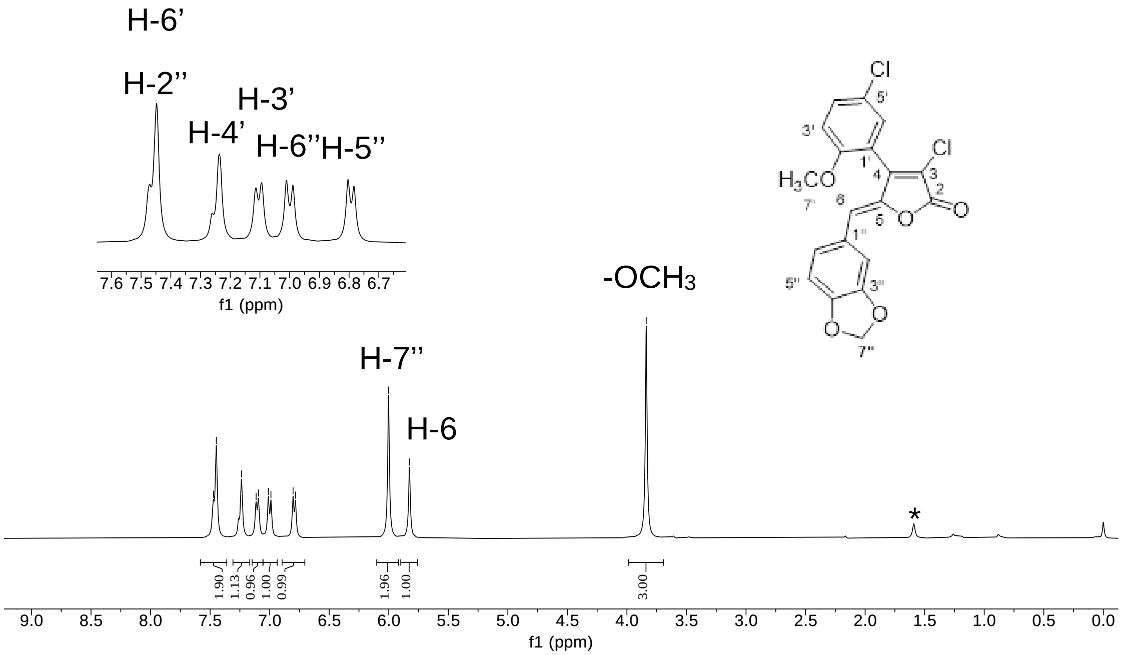


**Fig. S15** $^{1}H$ NMR (400 MHz, $CDCl_3$) of compound **15**. *Solvent residues and $H_2O$ signals.

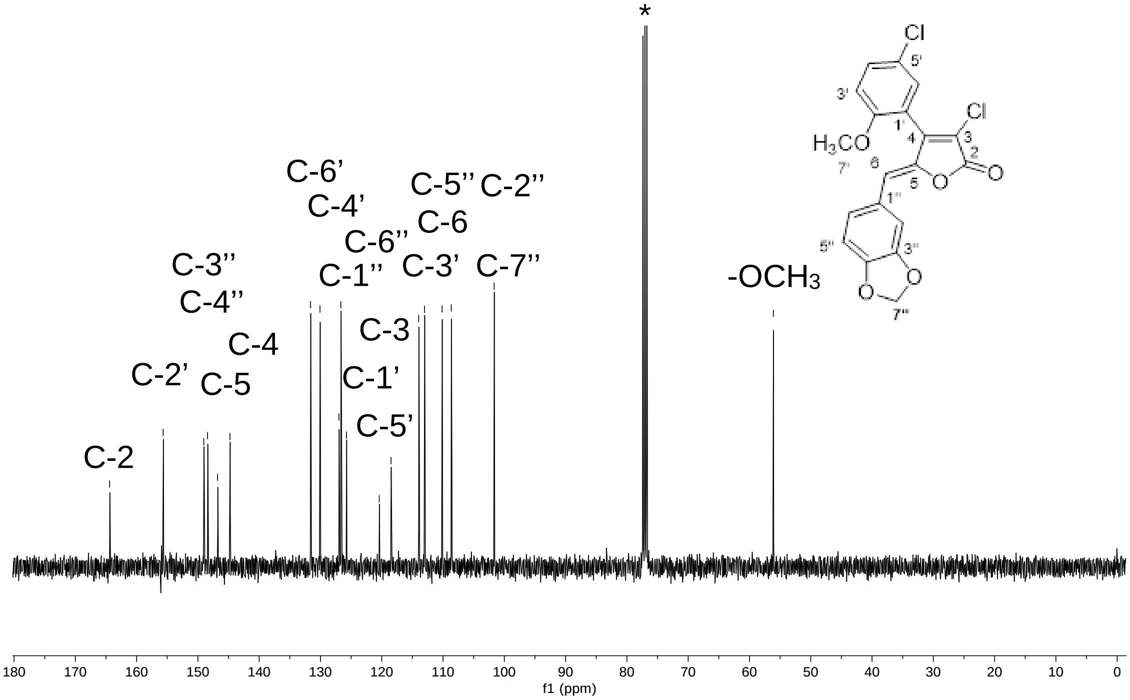


**Fig. S16** $^{13}C$ NMR (100 MHz, $CDCl_3$) of compound **15**. *Solvent residues signals.

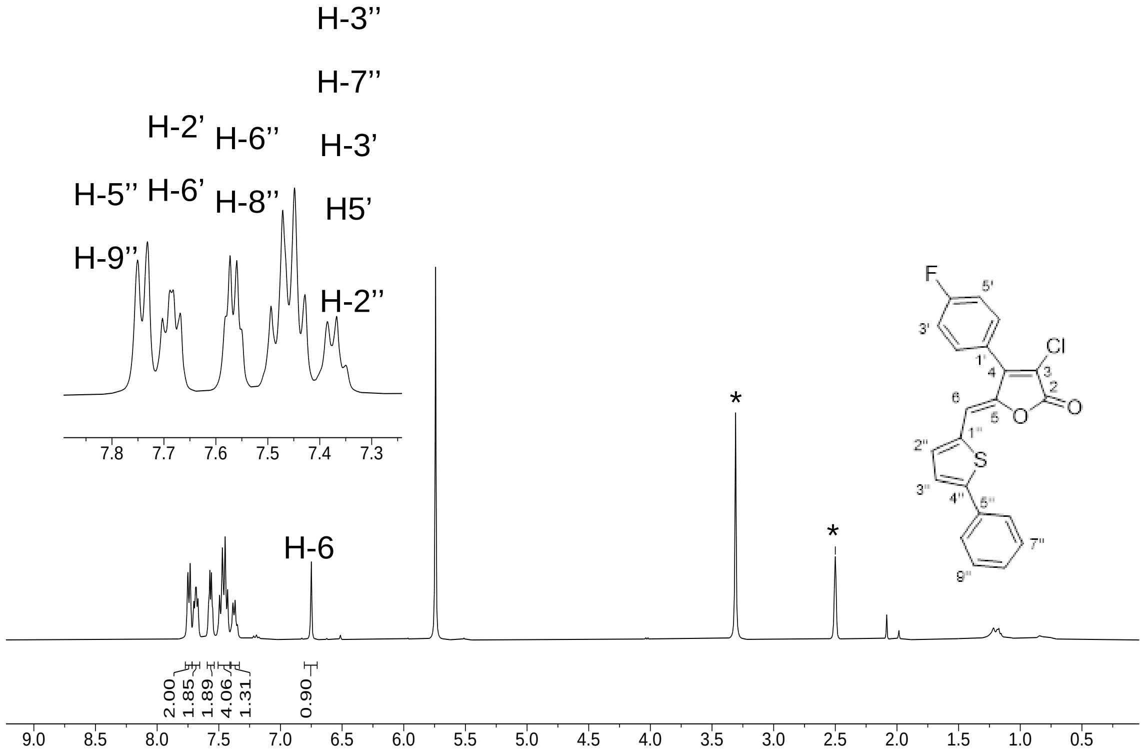


**Fig. S17** $^{1}H$ NMR (400 MHz, $(CD_3)_2SO$) of comp **16**. *Solvent residues and $H_2O$ signals.

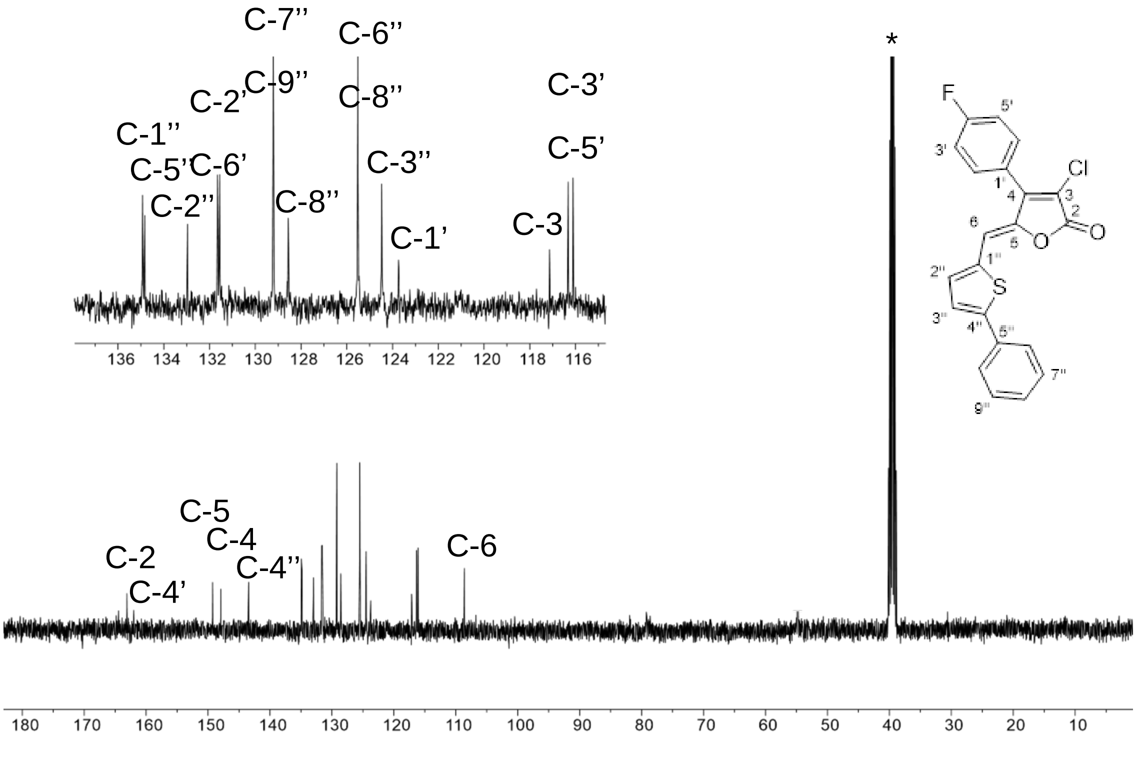


**Fig. S18** $^{13}C$ NMR (100 MHz, $(CD_3)_2SO$) of compound **16**. *Solvent residues signals.

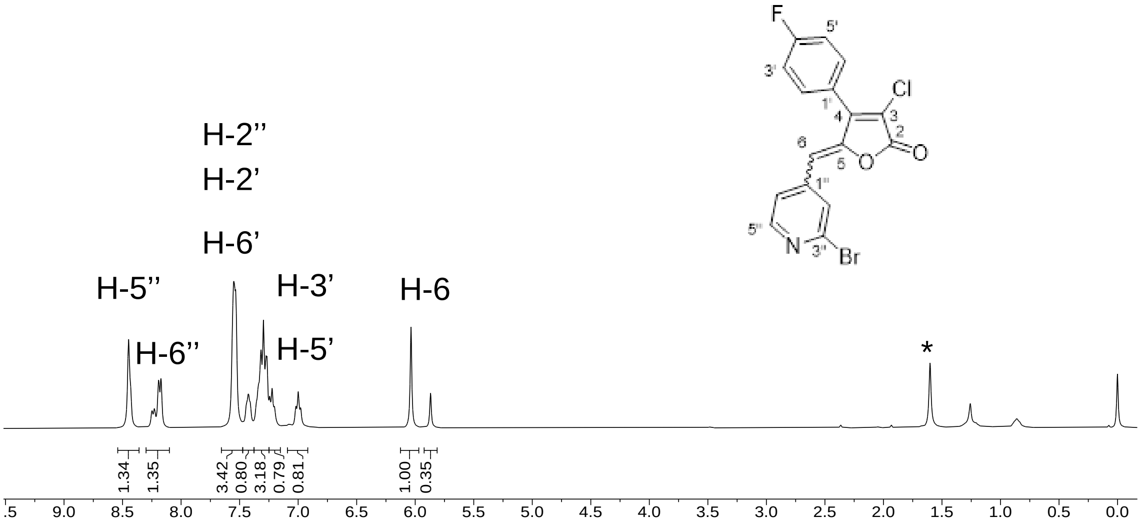


**Fig. S19** $^{1}H$ NMR (400 MHz, $CDCl_3$) of compound **17** (*Z/E* = 0.74/0.26). *Solvent residues and $H_2O$ signals.

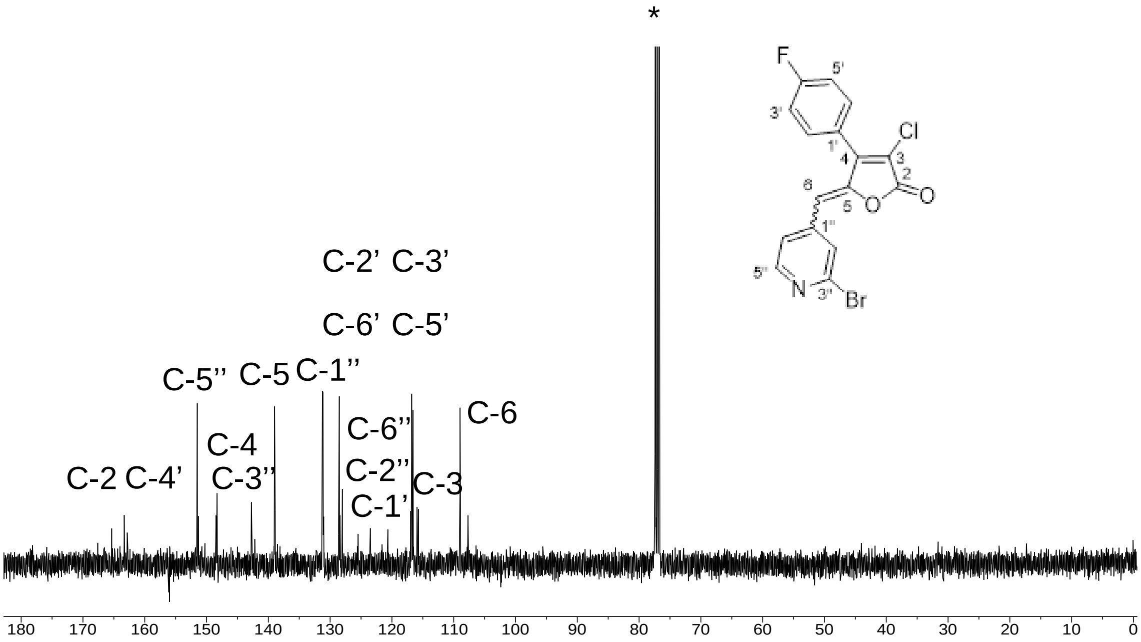


**Fig. S20** $^{13}C$ NMR (100 MHz, $CDCl_3$) of compound **17**. *Solvent residues signals.

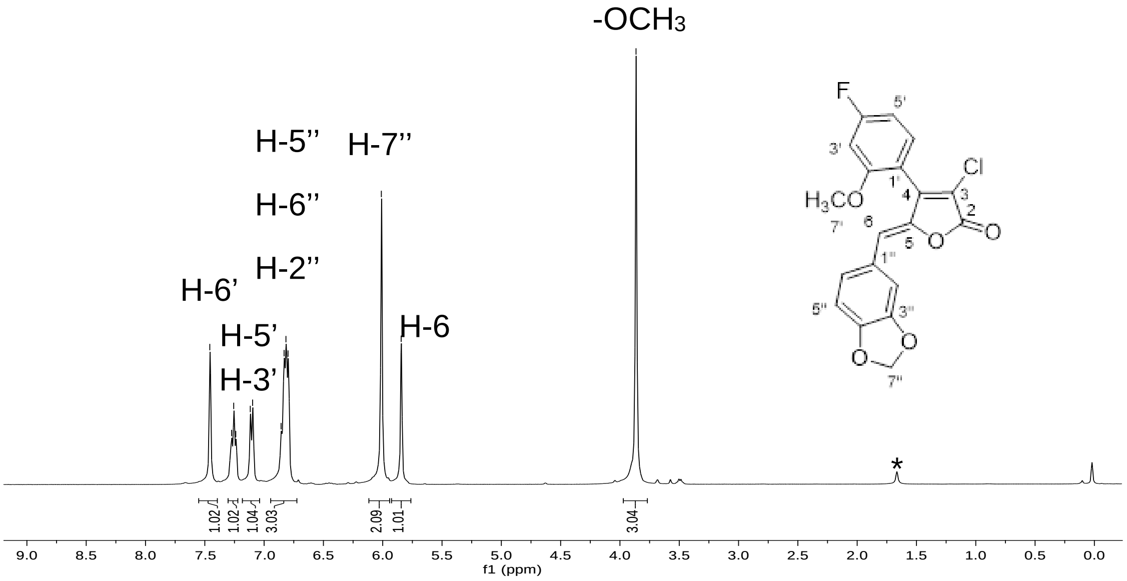


**Fig. S21** $^{1}H$ NMR (400 MHz, $CDCl_3$) of compound **18**. *Solvent residues and $H_2O$ signals.

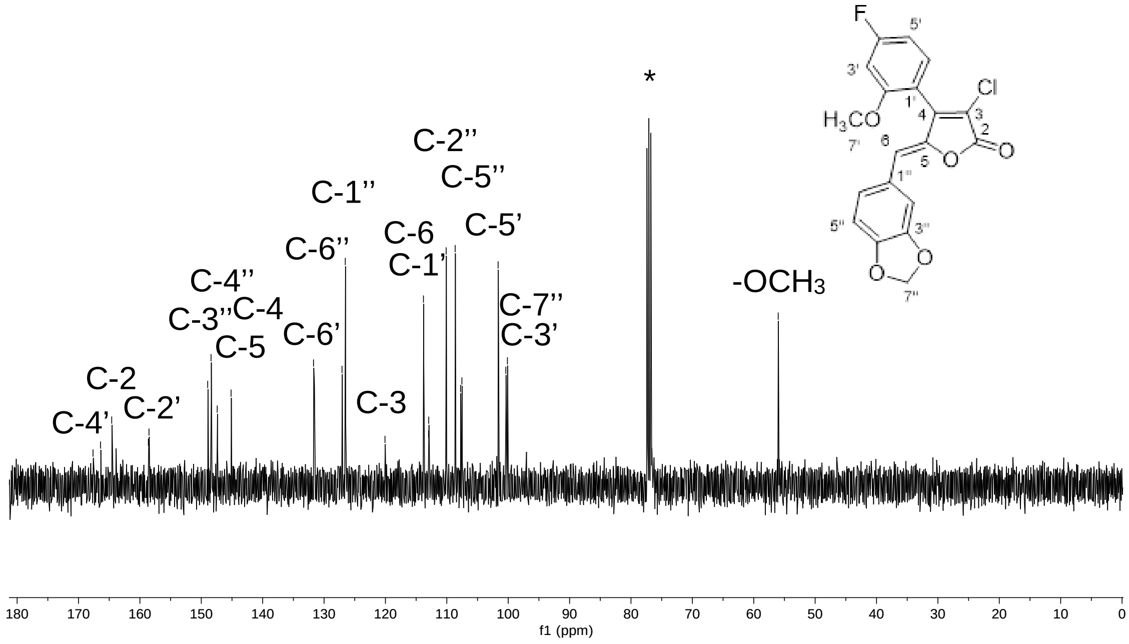


**Fig. S22** $^{13}C$ NMR (100 MHz, $CDCl_3$) of compound **18**. *Solvent residues signals.

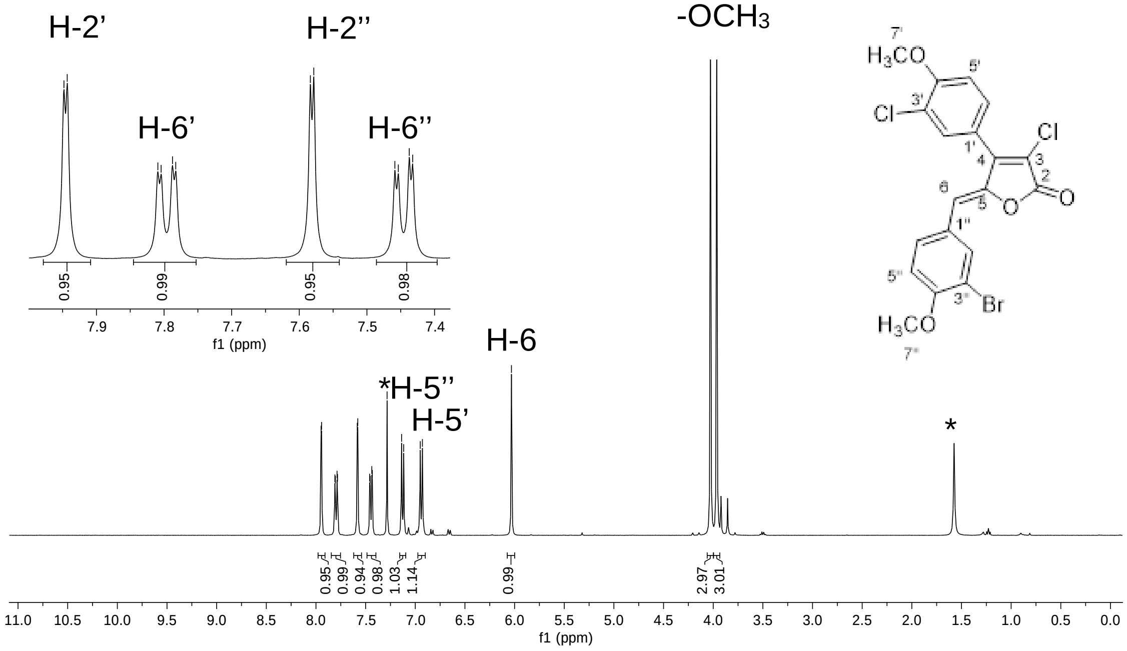


**Fig. S23** $^{1}$H NMR (400 MHz, $CDCl_3$) of compound **19** (*Z/E* = 96/4). *Solvent residues and $H_2O$ signals.

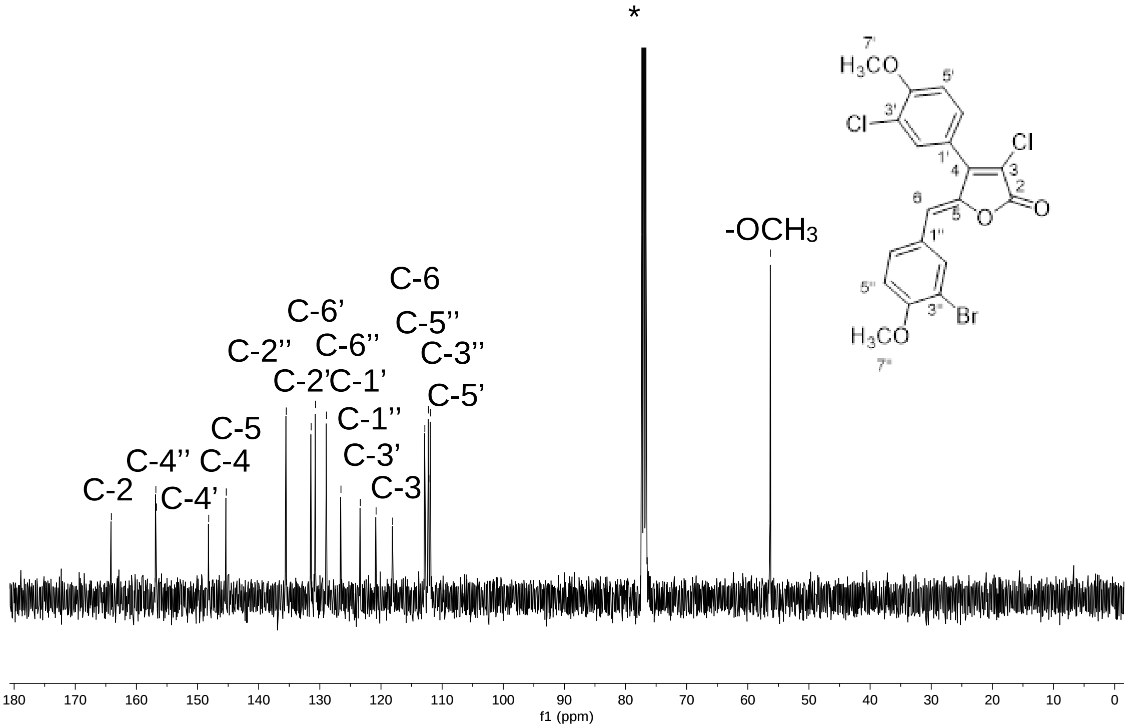


**Fig. 24** $^{13}$C NMR (100 MHz, $CDCl_3$) of compound **19**. *Solvent residues signals.

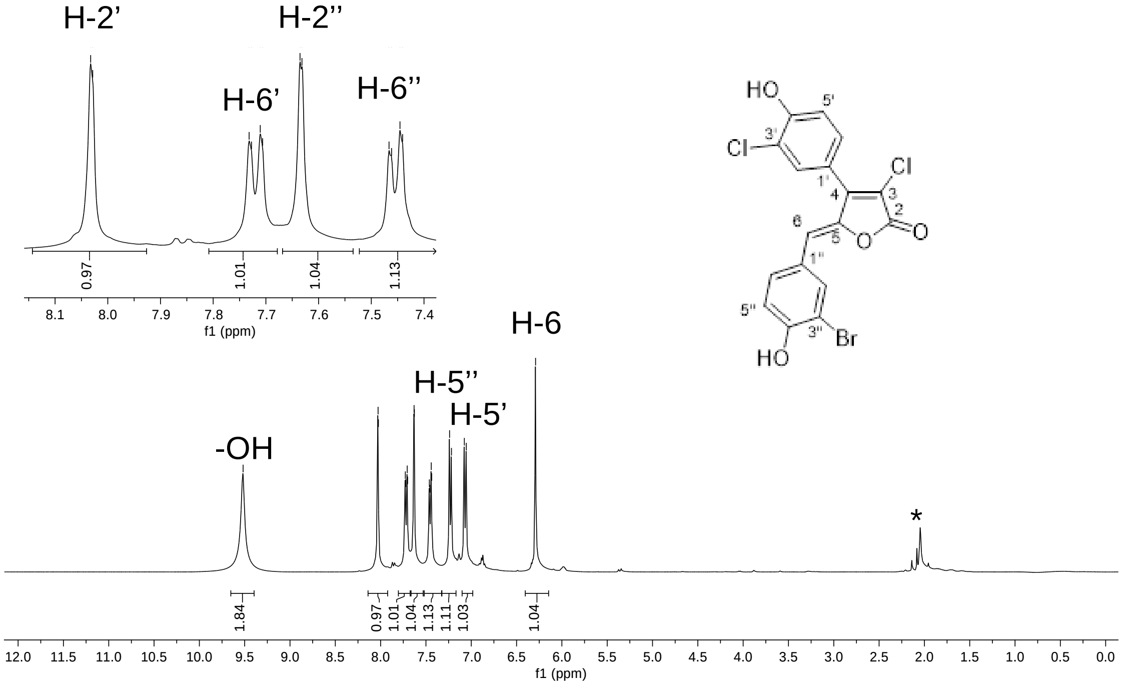


**Fig. S25** $^{1}$H NMR (400 MHz, $(CD_3)_2CO$) of compound **20**. *Solvent residues and $H_2O$ signals.

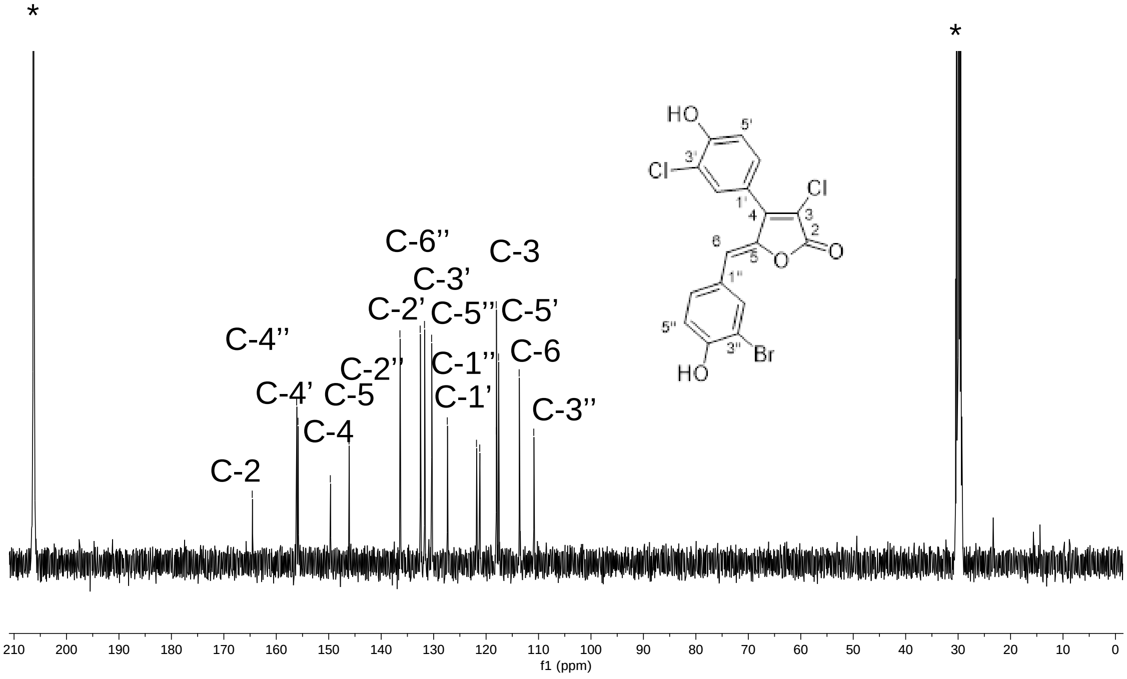


**Fig. S26** $^{13}$C NMR (100 MHz, $(CD_3)_2CO$) of compound **20**. *Solvent residue signals.

## 4. Physicochemical properties, lipophilicity and water solubility

**Table S1:** *In silico* parameters with data from Lipinski and water solubility for compounds **1-4**, **10**, **19**, and **20**.[4]

| Compounds | MW | Log $P_{o/w}$ (MLog*P*) | HBA | HBD | Log S (ESOL)* |
|---|---|---|---|---|---|
| **1** | 630.30 | 4.79 | 4 | 2 | -8.00 |
| **2** | 551.41 | 4.20 | 4 | 2 | -7.09 |
| **3** | 472.51 | 3.61 | 4 | 2 | -6.18 |
| **4** | 551.41 | 4.20 | 4 | 2 | -7.09 |
| **10** | 414.86 | 3.26 | 3 | 1 | -5.47 |
| **19** | 456.11 | 3.94 | 4 | 0 | -6.29 |
| **20** | 428.06 | 3.50 | 4 | 2 | -5.86 |

**MW:** molecular weight; **Mlog*P*:** log*P* calculated by the Moriguchi methodology;[5] **HBA:** hydrogen bond acceptors; **HBD:** hydrogen bond donors; **Log *S*:** topological method implemented from Delaney;[6] **ESOL:** *E*stimated *SOL*ubility.

*Solubility scale: (*Insoluble* < −10 *poorly* < −6 *moderately* < −4 *soluble* < −2 *very* < 0 < *highly*)

## 5. Molecular docking results

**Table S2:** Interaction energy calculations between PSII (D1) and synthezized compounds (**1–10**, **12–20**) and commercial herbicides lenacil and diuron.

| Protein (PDB: 3JCU) | Autodock Interaction energy (kcal $mol^{-1}$) | Swissdock Interaction energy (kcal $mol^{-1}$) |
|---|---|---|
| **1** | -9.3 | -7.14 |
| **2** | -9.2 | -7.01 |
| **3** | -9.4 | -7.67 |
| **4** | -9.3 | -7.87 |
| **5** | -9.5 | -7.93 |
| **6** | -9.6 | -7.90 |
| **7** | -9.5 | -8.78 |

| | | |
|---|---|---|
| **8** | -8.4 | -8.17 |
| **9** | -8.4 | -7.67 |
| **10** | -7.9 | -8.22 |
| **12** | -9.4 | -7.82 |
| **13** | -9.1 | -8.76 |
| **14** | -8.1 | -9.18 |
| **15** | -9.0 | -7.88 |
| **16** | -10.7 | -8.47 |
| **17** | -8.6 | -7.38 |
| **18** | -9.6 | -8.26 |
| **19** | -9.4 | -8.8 |
| **20** | -9.0 | -8.61 |
| Lenacil | -8.9 | -8.83 |
| Diuron | -7.0 | -8.50 |

**Table S3:** Docking results of most active compounds **2**, **4**, **10**, **20**

| **Protein (PDB: 3JCU)** | **Number of H-bonds (length)** | **Bond angle (°)** |
|---|---|---|
| **2** | 1 (2.013 Å) | 154 |
| **4** | 1 (2.305 Å) | 159 |
| **10** | 2 (2.216 Å & 1.82 Å) | 132 & 124.8 |
| **20** | 1 (2.142 Å) | 134 |
| Lenacil | 1 (1.924 Å) | 157 |

## 6. References


(1) Bellina, F.; Anselmi, C.; Martina, F.; Rossi, R. *Eur. J. Org. Chem.* **2003**,  (12), 2290-2302.

(2) Karak, M.; Acosta, J. A. M.; Barbosa, L. C. A.; Boukouvalas, J. *Eur. J. Org. Chem.* **2016**, *2016* (22), 3780-3787.

(3) Karak, M.; Barbosa, L. C. A.; Maltha, C. R. A.; Silva, T. M.; Boukouvalas, J. *Tetrahedron Lett.* **2017**, *58* (29), 2830-2834.

(4) Swiss institute bioinformatics (SwissADME), http://www.swissadme.ch/, accessed in August 2024.

(5) Moriguchi, I.; Hirono, S.; Liu, Q.; Nakagome, I.; Matsushita, Y. *Chem. Pharm. Bull.* **1992**, *40* (1), 127-130.

(6) Delaney, J. S. *J. Chem. Inf. Comput. Sci.* **2004**, *44* (3), 1000-1005.
